# Host genetic variability and pneumococcal disease: a systematic review and meta-analysis

**DOI:** 10.1186/s12920-019-0572-x

**Published:** 2019-09-13

**Authors:** Anne T. Kloek, Matthijs C. Brouwer, Diederik van de Beek

**Affiliations:** Department of Neurology, Amsterdam Neuroscience, Amsterdam UMC, University of Amsterdam, Meibergdreef, Amsterdam, The Netherlands

**Keywords:** Host genetic variability, Pneumococcal disease, Systematic review, Meta-analysis

## Abstract

**Background:**

Pneumonia, sepsis, meningitis, and empyema due to *Streptococcus pneumoniae* is a major cause of morbidity and mortality. We provide a systemic overview of genetic variants associated with susceptibility, phenotype and outcome of community acquired pneumococcal pneumonia (CAP) and invasive pneumococcal disease (IPD).

**Methods:**

We searched PubMed for studies on the influence of host genetics on susceptibility, phenotype, and outcome of CAP and IPD between Jan 1, 1983 and Jul 4, 2018. We listed methodological characteristics and when genetic data was available we calculated effect sizes. We used fixed or random effect models to calculate pooled effect sizes in the meta-analysis.

**Results:**

We identified 1219 studies of which 60 studies involving 15,358 patients were included. Twenty-five studies (42%) focused on susceptibility, 8 (13%) on outcome, 1 (2%) on disease phenotype, and 26 (43%) on multiple categories. We identified five studies with a hypothesis free approach of which one resulted in one genome wide significant association in a gene coding for lincRNA with pneumococcal disease susceptibility. We performed 17 meta-analyses of which two susceptibility polymorphisms had a significant overall effect size: variant alleles of *MBL2* (odds ratio [OR] 1·67, 95% confidence interval [CI] 1·04–2·69) and a variant in *CD14* (OR 1·77, 95% CI 1·18–2·66) and none of the outcome polymorphisms.

**Conclusions:**

Studies have identified several host genetics factors influencing risk of pneumococcal disease, but many result in non-reproducible findings due to methodological limitations. Uniform case definitions and pooling of data is necessary to obtain more robust findings.

**Electronic supplementary material:**

The online version of this article (10.1186/s12920-019-0572-x) contains supplementary material, which is available to authorized users.

## Background

Pneumococcal infection is a major cause of morbidity and mortality worldwide [1]. Invasive pneumococcal disease (IPD) is an infection confirmed by the isolation of *Streptococcus pneumoniae* from a normally sterile site, while non-invasive pneumococcal disease includes sinusitis, mastoiditis, acute otitis media, and community-acquired pneumonia (CAP). *Streptococcus pneumoniae* has been identified as the most common cause of CAP in adults [2–4]. In 2015, an estimated 515.000 deaths (range 302.000–609.000) were attributed to pneumococcal infection among children less than 5 years of age globally [5]. The incidence of IPD is strongly age-related, with the highest incidence in younger children and the elderly with incidence ranging from 11 to 27 per 100,000 in Europe [6–8]. Mortality rates for IPD vary from 12 to 22% in adults in the western world and are substantially higher in low income countries [7–11].

Pneumonia with empyema and/or bacteraemia, meningitis, and bacteraemia are the commonest manifestations of IPD. [12] Identified risk factors for IPD include splenectomy, cancer, and diabetes mellitus, but in a substantial proportion of patients no risk factor can be identified [7]. Extreme phenotype studies in patients with recurrent or familial IPD first identified genetic risk factors to increase susceptibility [13]. Most of the identified genetic variation was found in genes controlling the host response to microbes [14]. Subsequently several case–control and cohort studies described genetic variation to increase susceptibility and to predict unfavourable outcome of pneumococcal disease and disease phenotype [6, 9, 15].

In the past 20 years several genetic association studies investigated host genetics in relation to susceptibility and outcome of pneumococcal disease, sometimes showing conflicting results. Here we systematically review these studies, perform a meta-analysis and discuss the potential of these findings for understanding the pathophysiological mechanisms of pneumococcal disease.

## Methods

### Systematic review

We performed a systematic review and meta-analysis with the objective to summarize host genetic variation associated with susceptibility, phenotype or outcome of patients with IPD and CAP. The following search terms were used in PubMed: ((*Streptococcus pneumoniae*) OR (*S. pneumoniae*) OR pneumococcal OR pneumococcus) AND (polymorphisms OR polymorphism OR (genetic variant) OR (genetic variants) OR (genetic association study) OR (single nucleotide polymorphism) OR (single nucleotide polymorphisms) OR SNP OR SNPs OR genotype OR genotypes) without language restrictions and with search date cut offs between Jan 1, 1983 and Jul 4, 2018. We identified additional publications by checking the references in those published studies and via communicating with experts in the field. Extreme phenotype, review studies, and studies with specific patients groups like immunocompromised patients were excluded. Studies were eligible for inclusion if the population of interest was reported with at least one of the outcome measures.

### Meta-analysis and statistical analyses

Each study was scored for methodological quality, such as study design, definition of the investigated condition, ethnicity of included patients, sample size, selection of the control group, quality control of genotyping, statistical methods and correction for multiple testing. We performed meta-analyses for multiple studies that assessed a single genetic polymorphism (or a combination of polymorphisms) of which genotype data was available in the manuscript. Different nomenclatures of genetic variants included in the review can be found in Additional file 1: Table S1. Review Manager 5.3 was used to generate Forest plots and calculate overall effect sizes with a fixed effects model or random effects model if the results between studies were too heterogeneous (Q test for homogeneity *p* < 0.05) [16]. The funder of the study had no role in study design, data collection, data analysis, data interpretation, or writing of the report. The corresponding author had full access to all the data in the study and had final responsibility for the decision to submit for publication.

## Results

### Systematic review

**Fig. 1:**
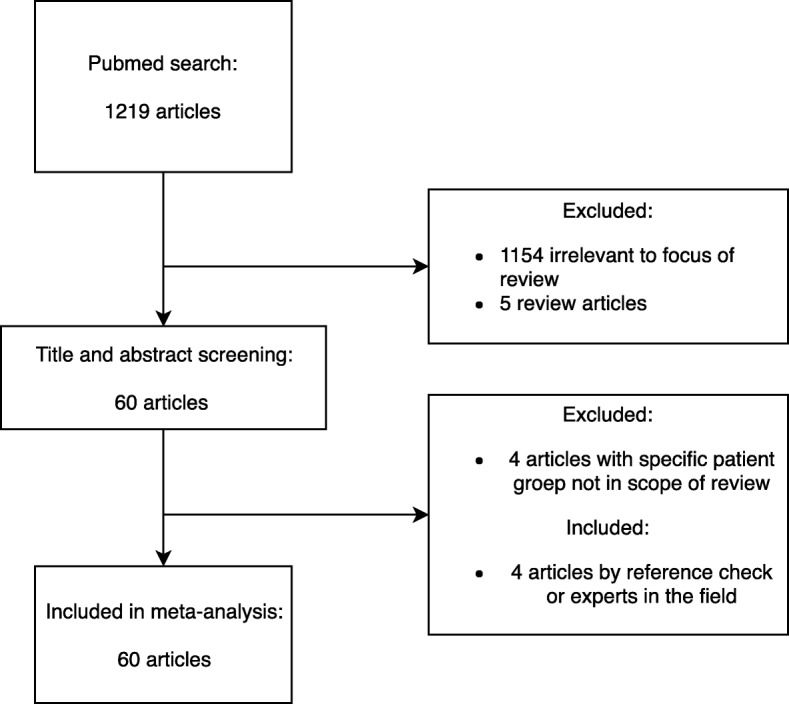
Flow diagram for study selection

The date of search was 4 July 2018 and yielded 1219 articles (Fig. 1 - flow diagram) of which 60 articles were eventually included in the review [17–76]. Studies were published from 2000 to 2018 and contained 16,034 patients included in 27 different cohorts from 15 countries. There was a substantial overlap of cohorts and patients between the published articles. Of all studies, 24 (40%) analysed the influence of genetic variation on susceptibility to pneumococcal disease, 8 (13%) on outcome, 2 (3%) on disease phenotype, and 26 (43%) studies assessed multiple categories of which 24 (40%) on susceptibility and outcome (Tables 1 and 2). Eight studies (13%) focused on patients with pneumococcal CAP, 49 studies (82%) on patients with IPD and 3 studies (5%) on IPD and pneumococcal CAP.

**Table 1:** Genetic-association studies on susceptibility to pneumococcal disease

Name, year, reference	Candi-date gene	Genetic variants*	Country of origin (*ethnicity)*	Patient groups	Patient -selection	N	Controls - selection	N	Results – Gene, genetic variation, risk allele/genotype: *p*-value, OR (95% CI) †
Pathogen recognition receptor signalling pathways									
Khor, 2007, [29] Cohort 1	*TIRAP*	31 variants	United Kingdom *(white)*	All ages with IPD	Blood, CSF, or joint fluid culture	191	Blood donors and cord blood samples	741	rs8177374 heterozygosity: *p* = 0.013, OR 0.65 (0.44–0.97)
Khor, 2007 Cohort 2	*TIRAP*	31 variants	United Kingdom *(unspecified*)	Pleural empyema	Empyema culture	36	Healthy adult blood donors	361	rs8177374 heterozygosity: *p* = 0.08, OR 0.74 (0.32–1.68)
Khor, 2007 Cohort 3	*TIRAP*	31 variants	Kenya *(African)*	Children with bacteraemia	Blood culture	164	Community-based	423	rs8177374 heterozygosity: *p* = 0.024, OR 0.30 (0.06–0.99)
Moens, 2007, [30]	*TLR2 TLR4*	rs5743703 rs5743704 rs5743708 rs4986790	Belgium *(white)*	All ages with IPD	Blood, CSF, or joint fluid culture	99	Family of hospital personnel, university employees	178	NS
Yuan, 2008, [33]	*TLR2 TLR4* *CD14*	rs5743708 rs4986790 rs4986791 rs2569190	Australia *(unspecified)*	Children with IPD	Blood culture	85	Healthy blood donors	409	- *TLR4* rs4986790/rs4986791 AG + GG/CT + TT genotypes: *P* < 0.05, OR 0.3 (0.1–1) - *CD14* rs2569190-CC genotype: *P* < 0.05, OR 1.7 (1–2.8)
Sanders, 2011, [43]	*TLR9*	rs5743836 rs352140	Netherlands *(white)*	Children and adolescents with BM	CSF culture	83	Healthy white adults without a known history of BM	392	NS
Van Well, 2013, [57]	*TLR2* *TLR4* *NOD1* *NOD2 CASP1*	rs5743708 rs4986790 rs6958571 rs2066844 rs2066845 rs2066847 rs2282659	Netherlands *(white)*	Children with BM	CSF culture	82	Ethnically matched healthy controls	1141	NS
Tellería-Orriols, 2013, [59]	*TLR2* *TLR 4 CD14*	rs5743708 rs4986790rs2569190	Spain *(white)*	Children with IPD	Culture of sterile site, PCR or antigen	114	Healthy White children	66	- *TLR2* rs5743708-GA + AA genotypes: *p* < 0.0001, OR 4.26 (2.19–8.3) - *CD14* rs2569190-CC: *p* = 0.0167, OR 1.93 (0.95–3.91)
Ellis, 2015, [63]	*IRAK4* *MYD88 IKBKG*	233 variants	United Kingdom *(white)*	All ages with IPD	Culture of sterile site	164	Geographically-matched population-based controls	164	*IRAK4* rs4251513 variant allele: *p* = 9.96 × 10^− 3^, OR 1.50 (1.10–2.04)
Carrasco-Colom, 2015, [65]	*IRAK4 IRAK1 IRAKM* *MYD88*	10 variants	Spain *(mixed, 92% white)*	Children with IPD and SIRS	Culture or PCR of sterile site	60	Patients with no previous immunodeficiency or IPD, nor concomitant infectious pathology	120	*P*-value in article adjusted by false discovery rate: not repoducible by re-calculation - *IRAK1* rs1059701-CC - *IRAK4* rs4251513-CC - *IRAK4* rs1461567-T - *MYD88* rs6853-AA
Gowin, 2017, [74]	*TLR2 TLR4 TLR9*	rs5743708 rs4696480 rs4986790 rs352140 rs5743836	Poland *(White)*	Children with BM	CSF culture or PCR	14	Family members	49	NS
Gowin, 2018, [76]	*TIRAP* *TLR2 TLR4 TLR9*	rs8177374 rs4696480 rs5743708 rs4986790 rs5743836 rs352140	Poland *(white)*	Children with bacterial meningitis	CSF culture or PCR	14	Family members	49	*- TIRAP* rs8177374 variant allele carriers: *p* = 0.0508, OR 4.5 (0.96–21.12) *- TIRAP* rs8177374 and MBL2 rs1800451 variant alleles cumulative effect: *p* = 0.035, OR = 4.9 (1.17–20.48)
Complement system									
Roy, 2002, [19]	*MBL2*	rs5030737 rs1800450 rs1800451 rs7096206	United Kingdom *(white)*	All ages with IPD	Sterile body site	337	Donors, neonates	1032	*MBL2* O/O genotype: *p* = 0.002, OR 2.59 (1.39–4.83)
Kronborg, 2002 [18]	*MBL2*	rs5030737 rs1800450 rs1800451 rs7096206	Denmark *(mixed, 97.9% white)*	Adults with IPD	Blood culture	140	Blood donors, laboratory personnel	250	NS
Moens, 2006, [27]	*MBL2*	rs5030737 rs1800450 rs1800451 rs7096206	Belgium *(white)*	All ages with IPD	Blood, CSF, or joint fluid culture	63	Sex-matched hospital employees, urology, and internal medicine outpatients	162	NS
Endeman, 2008, [35]	*MBL2*	rs5030737 rs1800450 rs1800451 rs7096206	Netherlands *(not specified)*	Adults with CAP	Blood / sputum culture	60	Blood bank donors	223	NS
Garcia-Laorden, 2008, [49]	*MBL* *MASP-2*	rs5030737 rs1800450 rs1800451 rs72550870	Spain (*white*)	Adults with CAP	Clinical symptoms and radiographic findings	195	Healthy control subjects, a control group of patients without relevant infectious diseases	1447, 519	NS
Garcia-Laorden, 2011, [44]	*SFTPA1* *SFTPA2* *SFTPD*	rs1059047 rs1136450 rs4253527 rs1059046 rs17886395 rs4253527 rs721917	Spain (*white*)	Adults with CAP	Clinical symptoms and radiographic findings and blood culture	326	Blood and bone marrow donors as well as hospital staff and patients without signs of relevant infectious diseases	1538	Associations below p < 0.05: - 5 haplotypes of *SFTPA1*, *SFTPA2* and *SFTPD*
Garcia-Laorden, 2012, [49] Cohort 1	*MBL2*	rs5030737 rs1800450 rs1800451	Spain *(white)*	Adults with CAP	Clinical symptoms and radiographic findings and blood culture	340	Blood and bone marrow donors, hospital staff and patients without signs of relevant infectious diseases	1736	NS
Garcia-Laorden, 2012 Cohort 2	*MBL2*	rs5030737 rs1800450 rs1800451	Spain *(unspecified)*	Adults with CAP	Blood, pleural fluid, sputum (+ bacterial recount or positive urinary antigen) culture	84	Healthy controls	91	NS
Brouwer, 2013, [58]	*MBL2*	rs5030737 rs1800450 rs1800451 rs7096206	Netherlands *(white)*	Adults with BM	CSF culture	299	Partners, non-related proxies	216	*MBL2* O/O genotype: *p* = 0.017, OR 8.21 (1.05–64.1)
Adriani, 2013, [56]	*C3 C5 C6 C7 C8B C9 CFH*	17 variants	Netherlands *(mixed, 94% white)*	Adults with BM	CSF culture	299	Partners, non-related proxies	216	NS after correction. *C7* rs13157656 dominant model: *p =* 0.04 OR 1.46 (1.02–2.09) *C3* rs1047286 recessive model *p =* 0.03 OR 3.14 (1.08–9.19)
Lundbo, 2014, [62]	*MBL2*	rs5030737 rs1800450 rs1800451 rs7096206	Scandinavia, Germany *(unspecified)*	Children with IPD	CSF, blood or other sterile site culture	1279	Age- and sex-matched	1263	NS
Mills, 2015, [64]	*MBL2*	rs5030737 rs1800450 rs1800451 rs7096206	United Kingdom *(unspecified)*	Sepsis in adults with CAP	Not specified	95	Individuals attending general practice surgeries for reasons other than infection	477	NS
Gowin, 2018, [76]	*MBL2* *CFH* *CFHR3*	rs5030737 rs1800450 rs1800451 rs1065489 rs3753396	Poland *(white)*	Children with BM	CSF Culture or PCR	14	Family members	49	*TIRAP* rs8177374 and *MBL2* rs1800451 cumulative effect*: p* = 0.035, OR = 4.9 (1.17–20.48)
Fcγ receptors									
Yee, 2000, [17]	*FCGR2A*	rs1801274	USA *(mixed)*	B-CAP (age not specified)	Blood or sputum culture	42	Randomly selected hospital patients	136	R131/R131 genotype: *p* < 0.05, OR 2.40 (1.18–4.87)
Yuan, 2003, [22]	*FCGR2A*	rs1801274	Australia *(unspecified)*	Children with sepsis	Blood culture, Ag in blood donors	63, 34	Children from vaccination programme/ Healthy blood donors	20, 57	R131/R131 genotype*: P* = 0.01, OR 2.81 (1.25–6.32)
Chapman, 2006, [25]	*PTPN22*	rs2476601	UK *(white)*	All ages with IPD	Culture of sterile body site	286	Ethnically matched	803	T allele: *P* = 0.004, OR = 1.56 (1.15–2.11)
Moens, 2006, [24]	*FCGR2A*	rs1801274	Belgium *(white)*	All ages with IPD	Blood, cerebrospinal fluid, or joint fluid culture	55	Sex-matched hospital employees, urology, and internal medicine outpatients	100	NS
Yuan, 2008, [33]	*FCGR2A*	rs1801274	Australia (*unspecified*)	Children with IPD	Blood culture	85	Healthy blood donors	409	R131/R131 genotype: *P* < 0.001, OR 2.46 (1.49–4.04)
Endeman, 2009, [37]	*FCGR2A*	rs1801274	Netherlands (*unspecified*)	Adults with CAP	Blood / sputum culture, urine antigen	60	Healthy unrelated Whites from the same geographical area	314	NS
Solé-Violán, 2011, [45]	*FCGR2A FCGR3A*	rs1801274 rs396991	Spain (*white*)	Adults with CAP and B-CAP	Blood culture, urine antigen	CAP = 319, B-CAP = 85	Unrelated healthy volunteers and patients without a previous history of relevant infectious diseases	1224	B-CAP *FCGR2A*– H131/H131 genotype: *p =* 0.01, OR 1.81 (1.09–2.43)
Bouglé, 2012, [52]	*FCGR2A*	rs1801274	France (*white*)	Adults with IPD	Culture of sterile site	243	ICU patients without infection	2789	NS
NFκβ signalling pathway									
Chapman, 2007, [32]	*NFKBIA NFKBIB NFKBIE*	43 variants (very rare excluded)	UK (*white*)	All ages with IPD	Blood, CSF, or joint fluid culture	288	Blood donors and cord blood samples	770	*NFKBIA* rs3138053 variant allele carriers: *p* = 0.0003, OR 0.60; (0.45–0.79) *NFKBIA* rs2233406 variant allele carriers: *p* = 0.00001, OR 0.55 (0.42–0.72)*NFKBIE* rs529948 variant allele carriers, *p* = 0.001, OR 0.59 (0.43–0.83)
Chapman, 2010, [38] Cohort 1	*NFKBIZ*	15 variants, (very rare excluded)	UK (*white*)	All ages with IPD	Culture from sterile site	275	Healthy adult blood donors, cord blood samples	163, 570	3 × 2 Chi-squared comparisons of genotypes, *p*-values below 0.05: rs600718: *p* = 0.01, rs616597: *p* = 0.001, rs685666: 0.036, rs6441627: 0.011, rs587555: *p* = 0.05, rs677011: 0.042, rs601225: *p* = 0.049
Chapman, 2010 Cohort 2	*NFKBIZ*	15 variants, (very rare excluded)	Kenya (*African*)	Children with IPD	Blood culture	173	Age and sex matched community-based	550	3 × 2 Chi-squared comparisons of genotypes: p-values below 0.05: rs600718: *p* = 0.022
Chapman, 2010, [40] Cohort 1	*NFKBIL2*	9 variants	UK (*white*)	All ages with IPD	Culture from sterile site	275	Healthy adult blood donors, cord blood samples	163, 570	Both cohorts: rs760477 heterozygosity: *p* = 0.0006, OR = 0.67 (0.53–0.84)rs4925858 heterozygosity: *p =* 0.003, OR = 0.70 (0.55–0.88)
Chapman, 2010 Cohort 2	*NFKBIL2*	9 variants	Kenya (*African*)	Children with IPD	Blood culture	173	Age and sex matched community-based	550	
Sangil 2018, [75]	*NFKBIA NFKBIE NFKBIL2 NFKBIZ*	10 variants	Spain (*white*)	Adults with IPD	Not specified	144	Ethnically matched	280	*NFKBIA* rs1050851-T: *p* = 0.04 *NFKBIE* rs2282151-C*: p* = 0.02*NFKBIZ*-CC rs645781: *p* = 0.02
Cytokines									
Schaaf, 2003, [21]	*IL10* *TNF* *LTA*	rs1800896 rs1800629 rs909253	Germany (*white*)	CAP and IPD (age not specified)	CSF, blood, pleural fluid, sputum culture	69	Unrelated age and sex-matched orthopaedic patients	50	NS
Schaaf, 2005, [23]	*IL6*	rs1800795	Germany (*white*)	CAP and IPD (age not specified)	CSF, blood, pleural fluid, sputum culture	100	Age matched	50	NS
Carrol, 2011, [42]	*IL-1Ra*	rs4251961	Malawi (*African*)	Children with IPD	Blood, sputum, CSF culture or Ag test or PCR	299	Healthy controls	933	NS
Martin- Loeches, 2012, [48]	*IL6*	rs1800795	Spain (*white*)	Adults with CAP	Blood culture, urine antigen	306	953 white Spanish unrelated healthy volunteers, 434 patients without a previous historyof relevant infectious diseases	1387	NS
Savva, 2016, [68]	*MIF*	rs5844572 rs755622	Netherlands *(white)*	Adults with BM	CSF culture	405	Partners, non-related proxies	329	NS
Sangil, 2018, [75]	*IL10* *IL12B* *IL1A* *IL1B ILR1 IL4*	33 variants	Spain (*white*)	Adults with IPD	Not specified	144	Ethnically matched	280	*IL1R1* rs3917254-CC: *p* = 0.04
Coagulation and fibrinolysis									
Benfield, 2010, [39]	*FVL*	rs6025	Denmark *(unspecified)*	Adults with IPD	Culture of CSF, blood or other sterile site	163	Age matched adults without infectious disease hospitalization	8147	NS
Mook, 2015, [66]	*CPB2* (TAFI)	rs1926447 rs3742264	Netherlands *(white)*	Adults with BM	CSF culture	716	Partners, non-related proxies	Not shown	NS
Other									
Roy, 2002, [20]	*CRP*	rs3138528	United Kingdom (*white*)	All ages with IPD	Blood, CSF, or joint fluid culture	205	Randomly selected local blood donors and transplant donors	345	Common allele: *P* = 0.001; OR 1.52 (1.18–1.96)
Chapman, 2007, [31]	*FCN2*	rs3124952 rs3124953 rs17514136 rs17549193 rs7851696	United Kingdom (*white*)	All ages with IPD	Blood, CSF, or joint fluid culture	290	Blood donors and cord blood samples	720	NS
Payton, 2009, [36]	*NOS2A*	9 variants	Malawi (*African*)	Children with IPD	Culture, PCR, antigen tests	229	Age matched	931	NS
Adriani, 2012, [51]	*ADRB2*	rs1042713 rs1042714	Netherlands (*mixed, 94% white*)	Adults with BM	CSF culture	396	Partners, non-related proxies	376	rs1042714 Gln/Glu genotype: *p* = 0.007, OR 1.52 (1.12–2.07)
Brouwer, 2012, [54]	*GLCCI1*	rs37972	Netherlands (*white*)	Adults with BM	CSF culture	699	Partners, non-related proxies	490	NS
Studies with genes in mixed categories									
Lingappa, 2011, [46]	34 genes	326 variants	USA *(European Americans (EA) and African Americans (AA))*	Children with IPD	Culture of sterile site	EA = 182 AA = 53	Bloodspot collection from new-borns, race/ethnicity and date of birth matched	361, 113	Associations below p < 0.05, none of the variants in both EA and AA): - In AA: 11 variants in 6 genes (*CD46, SFTPB, SFTPD, IL1B, ILIR1, PTAFR*) - in EA: 17 variants in 9 genes (*CD46, SFTPA1, SFTPD, IL1B, ILIR1, IL4, IL10, IL12B, FAS*)
Lundbo, 2015, [67]	*NFKBIE* *NFKBIA* *NFKBIL2* *NFKBIZ* *TIRAP* *PTPN22*	rs529948 rs3138053 rs2233406 rs760477 rs616597 rs8177374 rs2476601	Scandinavia, Germany *(unspecified)*	Children with BM/ bacteraemia	CSF or blood culture	372, 907	Age and sex matched	1273	Pneumococcal meningitis: *NFKBIE* rs529948 variant allele carriers, *p* = 0.0001, OR 1.68 (1.20–2.36) Combined patient groups: *NFKBIE* rs529948 variant allele carriers, *p* = 0.01, OR 1.24 (1.03–1.49) Other: NS
Hypothesis free studies									
Ellis, 2015, [63]	Sequencing of *IRAK4* *MYD88 IKBKG*	233 variants	United Kingdom *(white)*	All ages with IPD	Culture of sterile site	164	Geographically-matched population-based	164	*IRAK4* rs4251513 variant allele: *p =* 9.96 × 10^−3^, OR = 1.50 (1.10–2.04)
Ferwerda, 2016, [72]	Sequencing of 46 genes	1854 variants	Netherlands (*white*)	Adults with BM	CSF culture	435	Partners, non-related proxies	416	*CARD8* rs2008521-T allele: *p* = 8.2 × 10^−4^, OR 1.82 (1.28–2.75) *CXCL1* rs56078309-A allele: *p* = 8.2 × 10^− 4^, OR 1.96 (1.34–2.87)
Kenyan Bacterae-mia Study Group, 2016, [70]	GWAS	787,861 variants, 10 million variants after imputation	Kenya (*African*)	Children with bacteraemia	Blood culture	429 113	Sex, ethnic group, and geographic area matched controls	2677 1136	17 variants above genome-wide significance (*p* < 5 × 10^−8^). Strongest association in discovery cohort: (minor allele = risk allele) LincRNA rs14081715- additive model: p imputed = 7.25 × 10^−9^, OR = 2.74 Replication cohort: LincRNA rs14081715- additive model: *p* = 0.001, OR 2.72
Kloek, 2016, [71]	Exome array analysis	102,097 variants	Netherlands (*white*)	Adults with BM	CSF culture or PCR	469	Population-based controls	2072	*COL11A1* rs139064549-G allele: *p* = 1.51 × 10^−6^, OR 3.21 (2.05–5.02) *EXOC6B* rs9309464-G allele: *p* = 6.01 × 10^−5^, OR 0.66 (0.54–0.81)

Abbreviations: *Ag* agglutination*,* *BM* bacterial meningitis, *B-CAP* bacterial-CAP, *CAP* community acquired pneumoniae, *CI* confidence interval, *CSF* cerebrospinal fluid, *GWAS* genome wide association study, *IPD* invasive pneumococcal disease, *NS* not significant, *OR* odds ratio, *PCR* polymerase chain reaction, *PM* pneumococcal meningitis

*Genetic variants: Synonyms of genetic variants can be found in Supplementary Table 1. ^†^ Results: None of the *p*-values are corrected for multiple testing

**Table 2:** Genetic-association studies on outcome and phenotype of pneumococcal disease

Name, year, PMID	Candidate gene	Genetic variants*	Country of origin (*ethnicity*)	Patient groups	N	Patients- Selection criteria	Outcome measures -(% mortality, adverse events)	Results – Gene, genetic variation, risk allele/genotype: *p*-value, OR (95% CI) ^†^
Pathogen recognition receptor signalling pathways								
Van Well, 2012, [53]	*TLR2 TLR4 TLR9 NOD1 NOD2 CASP1 TRAIL*	11 variants	Netherlands *(white)*	Children with BM	66	CSF culture	Hearing loss (21%)	- *TLR9* rs5743836 TC and CC genotypes: *p* = 0.023, OR 2.5 (1.1–5.4) and *p* = 0.017, OR 5.0, (1.4–17.4)Combined carriership: - TLR2 rs5743708 and TLR4 rs4986790 AG genotype: p = 0.03, OR 13.9 (1.3–147)- TLR4 rs4986790 and TLR9 rs5743836 mutant alleles: *p* = 0.003, OR 6.0 (1.7–21.3)
Garnacho-Montero, 2012, [50]	*TLR2 TLR4*	rs5743708 rs4986790 rs4986791	Spain *(white)*	Adults with sepsis	117	Sterile site and BAL/tracheal aspirate culture	Septic shock (34%) In-hospital mortality (18.8%) 90 day mortality (21.4%)	NS
Carrasco-Colom, 2015, [65]	*IRAK4* *IRAK1 IRAKM MYD88*	10 variants	Spain *(mixed, 92% white)*	Children with IPD and SIRS	60	Sterile site culture or PCR	Pleuro-pneumonia (7%) Sequelae (33%) Mortality (3%) Serotypes	- Pleuropneumonia: *IRAKM* rs1624395-G and rs1370128-C; *p* = 0.0147, OR 1.83 (1.23–2.74) and *p* = 0.0055, OR 2.06 (1.37–3.11) - Sequelae: *IRAK4* rs4251513-nonGG: *p* = 0.0010, OR 7.07 (2.64–18.87)- Death: *MYD88* rs6853-nonAA and rs6853-G: *p* = 0.0054, OR 16.09 (3.34–77.57) and *p* = 0.0064, OR 8.39 (2.47–28.46) - Serotypes: NS
Complement system								
Kronborg, 2002, [18]	*MBL2*	rs7096206 rs5030737 rs1800450 rs1800451	Denmark *(mixed, 97.9% white)*	Adults with IPD	140	Blood culture	Outcome (not specified, mortality of 17%)	NS
Perez, 2006, [26]	*MBL-2*	rs5030737 rs1800450 rs1800451	Spain *(unspecified)*	Adults with CAP	97	Blood or pleural fluid culture/ sputum culture + positive Ag test or quantitative bacterial count	Bacteraemia (53%) Risk class of mortality (Fine scale) I (15%), II (11%), III (17%), IV (42%), V (15%)	- Bacteraemia: *MBL2* AA genotype: *p* = 0.02, OR 2.74 (1.01–7.52)- Risk class mortality: NS
Endeman, 2008, [35]	*MBL2*	rs5030737 rs1800450 rs1800451 rs7096206	Netherlands *(unspecified)*	Adults with CAP	60	Blood or sputum culture	Outcome - ICU admission (11%), length of hospital stay (median 11 (range 2–153)	NS
Garcia-Laorden, 2008, [34]	*MBL* *MASP-2*	rs5030737 rs1800450 rs1800451 rs7096206 rs72550870	Spain (*white*)	Adults with CAP	195	Clinical symptoms and radiographic findings	Severe sepsis (16%), septic shock (14%) ICU admission (22%), MODS (10%), high pneumonia severity index (59%), bacteraemia (8%), ARF (70.9%), ARDS (5%), 90 day mortality (9%)	NS
Woehrl, 2011, [47]	*C3 C5 C6 C7 C8B C9 CFH*	17 variants	Netherlands *(white)*	Adults with BM	217	CSF culture	Outcome - unfavourable (24%) vs favourable (76%, GOS 5)	*C5* rs17611-GG genotype: *p* = 0.002, OR 2.25 (1.33–3.81)
Garcia-Laorden, 2012, [49]	*MBL2*	rs5030737 rs1800450 rs1800451 rs7096206	Spain *(white)*	Adults with CAP	346	Clinical symptoms and radiographic findings and blood culture	Severity sepsis, ICU admission (38%), acute renal failure (33%), MODS (21%), high pneumonia severity index (56%), bacteraemia (28%), ARF (72%), ARDS (8%), 90 day mortality (7%)	NS
Garnacho-Montero, 2012, [50]	*MBL-2*	rs1800450 rs1800451 rs5030737	Spain *(white)*	Adults with sepsis	117	Culture of sterile site	Septic shock (34%) In-hospital mortality (19%) 90 day mortality (21%)	*MBL2* AO/OO variants: - Septic shock: aHR 15.3 (3.5–36.5)- In hospital mortality: aHR 3.2 (1.01–9.8) - 90 day mortality: aHR 2.2, (1.1–8.1)
Brouwer, 2013, [58]	*MBL2*	rs5030737 rs1800450 rs1800451 rs7096206	Netherlands *(white)*	Adults with BM	299	CSF culture	Septic shock, systemic complications Mortality (8%), - unfavourable outcome: GOS 1–4 (28%) Serotypes	NS
Lundbo, 2014, [62]	*MBL2*	rs5030737 rs1800450 rs1800451 rs7096206	Scandinavia, Germany *(unspecified)*	Children with IPD	1279	CSF, blood or other sterile site culture	Mortality (2%) and serotypes	NS
Muñoz-Almagro, 2014, [61]	*MBL2*	rs5030737 rs1800450 rs1800451 rs7096206 rs11003125 rs7095891	Spain *(mixed)*	All ages with IPD	147	CSF, blood, sterile body site culture or PCR	Serotypes, children < 18 years (69%) vs adults (31%)	- Children < 2 years vs other *MBL2* O/O and XA/O: *p* = 0.031- Children < 2 years vs other (opportunistic or low-attack-rate serotypes only) *MBL2* O/O and XA/O: *p* = 0.02
Mills, 2015, [64]	*MBL2*	rs5030737 rs1800450 rs1800451 rs7096206	United Kingdom *(unspecified)*	Sepsis in adults with CAP	245	Not specified	28-day mortality from CAP sepsis (19%)	NS
Kasan-moentalib, 2017, [73]	*MASP-2*	rs2273346 rs12711521 rs12142107 rs139962539	Netherlands *(white)*	Adults with BM	397	CSF Culture	Unfavourable outcome: GOS scale 1–4 (32%)	NS
Fcγ receptors								
Solé-Violán, 2011, [45]	*FCGR2A FCGR3A*	rs1801274 rs396991	Spain (*white)*	Adults with CAP and B-CAP	CAP:319, B-CAP:85	Blood culture, urine antigen	Acute renal failure (32%), ARDS (8%), severe sepsis (41%), 28 (4%) and 90 day (6%) mortality	Bacteraemic vs non-bacteraemic CAP: *FCGR2A*-H/H: *p* = 0.00016, OR 2.9 (1.58–5.3) B-CAP and CAP: - Acute renal failure *FCGR2A*-H/H: *p* = 0.004, OR 2.32 - Acute respiratory stress syndrome *FCGR2A*-H/H: *p* = 0.047, OR 2.17- Severe sepsis *FCGR2A*-H/H: *p* = 0.037. OR 1.8
Garnacho-Montero J, 2012, [50]	*FCGR2A*	rs1801274	Spain (*white*)	Adults with sepsis	117	Culture of sterile site	Septic shock (34%) In-hospital mortality (19%) 90 day mortality (21%)	NS
Bouglé, 2012, [52]	*FCGR2A*	rs1801274	France (*white*)	Adults with IPD	243	Culture of sterile site	Hospital mortality (31%)	Hospital mortality *FCGR2A*-H/H: *p* = 0.004, OR 0.251 (0.098–0.645)
NFκβ signalling pathway								
Chapman, 2010, [38] Cohort 1	*NFKBIZ*	15 variants, (very rare excluded)	UK (*white*)	All ages with IPD	275	Culture from sterile site	Outcome (not specified)	NS
Chapman, 2010 Cohort 2	*NFKBIZ*	15 variants, (very rare excluded)	Kenya (*African*)	Children with IPD	173	Blood culture	Outcome (not specified)	NS
Chapman, 2010, [40] Cohort 1	*NFKBIL2*	9 variants	UK (*white*)	All ages with IPD	275	Culture from sterile site	Mortality (10%)	NS
Chapman, 2010 Cohort 2	*NFKBIL2*	9 variants	Kenya (*African*)	Children with IPD	173	Blood culture	Mortality (28%)	NS
Geldhoff, 2013, [55]	*CARD8 NLRP1* *NLRP3*	rs2043211 rs11621270 rs35829479	Netherlands (*white*)	Adults with BM	531 (72% PM)	CSF culture	Mortality (18%), unfavourable outcome: GOS 1–4 (38%), systemic complications, neurological complications	*CARD8* rs2043211-TT genotype: - Unfavourable outcome: *p* = 0.018, OR 2.19 (1.15–4.81) - Systemic complications: *p* = 0.016, OR 2.48 (1.29–4.7) - Neurological complications: *p =* 0.022, 3.03 (1.34–6.85) *NLRP1* rs11651270-TT genotype: - Mortality*: p* = 0.047, OR 1.97 (1.02–3.85)
Cytokines								
Schaaf, 2003, [21]	*IL10* *TNF* *LTA*	rs1800896 rs1800629 rs909253	Germany (*white*)	CAP and IPD (age not specified)	69	CSF, blood, pleural fluid, sputum culture	Septic shock (19%), complications (48%), mortality (7%)	*IL10*-GG genotype: - severity (development of septic shock)*: p* = 0.008, OR 6.1 (1.4–27.2) - complications and mortality: NS (re-calculated)
Schaaf, 2005, [23]	*IL6*	rs1800795	Germany (*white*)	CAP and IPD (age not specified)	100	CSF, blood, pleural fluid, sputum culture	Bacterial dissemination (25%)	GG genotype*: p* = 0.04, OR 0.26 (0.07–0.94)
Carrol, 2011, [42]	*IL-1Ra*	rs4251961	Malawi (*African*)	Children with IPD	299	Blood, sputum, CSF culture or Ag test or PCR	Mortality (22%)	NS
Doernberg, 2011, [41]	*MIF*	rs5844572rs755622	USA, Germany (*white*)	Adults with IPD	30, 89	Culture from sterile body site	Disease phenotype: meningitis (14%)	Meningitis vs no meningitis: - rs5844572–77 and 7X genotypes: *p* = 0.02, OR = 3.34 (1.34–8.35)
Martin- Loeches, 2012, [48]	*IL6*	rs1800795	Spain (*white*)	Adults with CAP	306	Blood culture, urine antigen	ARDS (7%), septic shock (20%), multiple organ dysfunction syndrome (18%), hospital mortality (6%)	GG genotype: - ARDS: *p* = 0.002, OR = 0.25 (0.07–0.79) - septic shock: *p* = 0.006, OR = 0.46 (0.18–0.79) - multiple organ dysfunction syndrome*: p* = 0.02, OR = 0.53 (0.27–0.89)- survival (adjusted for age, gender, comorbidity, hospital of origin, and PSI): *p* = 0.048, OR = 0.27 (0.07–0.98)
Savva, 2016, [68]	*MIF*	rs5844572 rs755622	Netherlands *(white)*	Adults with BM	405	CSF culture	Unfavourable outcome- GOS 1–4 (33%), mortality (7%)	Unfavourable outcome: - rs5844572–77 and 7X: *p* = 0.005, OR 1.89 (1.21–2.96) - rs755622- GC and CC: *p* = 0.003, OR 1.9 (1.24–2.92) Mortality:- rs5844572–77 and 7X: *p* = 0.03, OR 2.27 (1.07–4.83) - rs755622 - GC and CC*: p* = 0.01, OR 2.6 (1.01–3.78)
Coagulation and fibrinolysis								
Benfield, 2010, [39]	*FVL*	rs6025	Denmark *(unspecified)*	Adults with IPD	163	Culture of CSF, blood or other sterile site	Mortality (15%), ICU admission	NS
Brouwer, 2014, [60]	*SERPINE1* (PAI-1)	rs1799889	Netherlands *(white)*	Adults with BM	400	CSF culture	Unfavourable outcome- GOS 1–4 (33%), mortality (8%), cerebral infarction 14%), haemorrhages (2%)	5G/5G genotype (low expression): - Unfavourable outcome: *p* = 0.035, OR 1.69 (1.03–2.78) - Mortality: *p* = 0.039 OR 2.23 (1.02–4.86) - Cerebral infarction: *p* = 0.011, OR 2.20 (1.19–4.07) - Haemorrhages: p = 0.005, OR 9.94 (1.89–52.17)
Mook, 2015, [66]	*CPB2* (TAFI)	rs1926447 rs3742264	Netherlands *(white)*	Adults with BM	716	CSF culture	Unfavourable outcome – GOS 1–4 (29%), death (7%), systemic complications (31%)	Unfavourable outcome and death: NSSystemic complications: - rs3742264 -AA allele vs common allele: *p* = 0.008, OR 0.40 (0.20–0.79)
Other								
Eklund 2006, [28]	*CRP*	rs1800947 rs2794521 rs1130864	Finland (*white*)	Patients with bacteraemia	42	Blood culture	Mortality (19%)	rs2794521- GG homozygotes*: p* = 0.03, OR 9.6 (1.3–72.5) *recalculated*
Payton, 2009, [36]	*NOS2A*	9 variants	Malawi (*African*)	Children with IPD	229	Culture, PCR, antigen tests	Mortality (22%)	NS
Adriani, 2012, [51]	*ADRB2*	rs1042713 rs1042714	Netherlands (*mixed, 94% white*)	Adults with BM	396	CSF culture	All BM unfavourable outcome: GOS 1–4 (23%), mortality (7%)	NS
Brouwer, 2012, [54]	*GLCCI1*	rs37972	Netherlands (*white*)	Adults with BM	699	CSF culture	Treatment effect dexamethasone (mortality)	NS
Studies with genes in mixed categories								
Lundbo, 2015, [67]	*NFKBIE* *NFKBIA* *NFKBIL2* *NFKBIZ* *TIRAP* *PTPN22*	rs529948 rs3138053 rs2233406 rs760477 rs616597 rs8177374 rs2476601	Scandinavia, Germany *(unspecified)*	Children with BM / bacteraemia	372, 907	CSF or blood culture	30 day mortality (2%)	NS
Hypothesis free studies								
Valls Seron, 2016, [69]	Exome array analysis	24,000 variants	Netherlands (*white*)	Adults with BM	472	CSF culture	Unfavourable outcome: GOS 1–4 (32%), mortality (8%)	*- AKT3* rs10157763 –A allele: *p* = 9.9 × 10^− 5^, OR 1.88 (1.4–2.6) *- RAET1E* rs3798763 and rs6925151 –G allele: *p* = 9.4 × 10^− 5^, OR 1.9 (1.4–2.6)- *DCTN4* rs11954652 and rs6869603 –G allele: *p* = 2.4 × 10^− 5^, OR 5.6 (2.4–12.9)
Ferwerda, 2016, [72]	Sequencing of 46 genes	1385 variants	Netherlands (*white*)	Adults with BM	435	CSF culture	Unfavourable outcome: GOS 1–4 (34%), mortality (8%)	*- IRAK4* rs4251552 –G allele: *p* = 4.8 × 10^− 4^, OR 2.86 (1.58–5.18)*- NOD2* rs2067085 –G allele: *p* = 5.1 × 10^− 4^, OR 2.16 (1.40–3.34)

Abbreviations: *Ag* agglutination, *aHR* adjusted Hazard ratio, *ARDS* acute respiratory stress syndrome, *ARF* Acute respiratory failure, *BM* bacterial meningitis, *B-CAP* bacterial-CAP, *CAP* community acquired pneumoniae, *CI* confidence interval, *CSF* cerebrospinal fluid, *GOS* Glasgow Outcome Scale, *GWAS* genome wide association study, *ICU* Intensive care unit, *IPD* invasive pneumococcal disease, *MODS* Multiple organ dysfunction syndrome, *NS* not significant, *OR* odds ratio, *PCR* polymerase chain reaction, *PM* pneumococcal meningitis

*Genetic variants: Synonyms of genetic variants can be found in Additional file 1: Table S1. † Results: None of the *p*-values are corrected for multiple testing

Twenty-eight studies (47%) were performed in adults (8188 patients) and 15 studies (25%) in children (4988 patients), 13 (22%) in all age categories (2675 patients) and 4 studies (7%) did not specify the age range of included patients. The population was limited to white patients in 39 studies (64%), mixed ethnicity in 9 studies (15%), and African origin in 3 studies (5%); ethnicity was not specified in 9 studies (15%). The sample size was less than 100 patients in 17 studies (28%), 100–500 patients in 40 studies (67%), and more than 500 in 3 studies (5%). The study population was defined by positive cultures of blood, cerebrospinal fluid or joint fluid in 41 studies (68%), and in 2 studies (3%) cultures of sputum or tracheal aspirate were included as well. Other studies used PCR, antigen tests or both (14 studies, 23%) to confirm bacterial presence. The control populations of the 57 susceptibility cohorts varied considerably and included healthy population-based controls, blood donors, participants in vaccine programs, patients from other hospital departments, university personnel or proxies and family members of patients. Some studies did specify if controls were ethnically, age or sex matched (32 cohorts, 56%).

Most studies (92%) had a candidate genetic variant approach looking at a selection of single nucleotide polymorphisms (range 1 to 326 polymorphisms; median 4). Five studies had a hypothesis free approach, including 1 genome wide association study, 2 exome wide association studies, and 2 sequencing studies [63, 69–72]. Most studies (41; 68%) determined genotypes by PCR followed by various methods of allelic discrimination, of which 18 studies confirmed genotypes with sequencing, 3 studies with retesting of samples and 19 studies did not mention if or how genotypes were confirmed. Eleven studies (18%) used real time PCR (by Taqman® genotyping assays), 1 (2%) PCR with mass spectrometry analysis, and 7 (12%) next generation sequencing (12%) for determination of genotypes. Seven studies (12%) described blinding of laboratory personnel for the clinical information.

The χ^2^ test and/or Fisher’s exact test was used in 48 studies (80%) to compare genotypes of selected groups. Logistic regression with correction for confounders to compare genotype frequencies between selected groups was done in 31 studies (52%). Correction for multiple testing was used in 23 (66%) of the 35 studies that assessed three or more polymorphisms.

### Meta-analysis

Meta-analysis could be done for 16 (combinations of) polymorphisms assessing an association with susceptibility and for 1 combination of polymorphisms assessing an association with outcome of pneumococcal disease. The number of cohorts in the meta-analysis varied between 2 and 10. Significant heterogeneity was found in 8 studies included in the meta-analyses for which therefore a random-effects model was used. Forest plots were made and overall ORs with 95% CIs were calculated (Additional file 2).

### Candidate gene approach

#### Pathogen recognition receptor signalling pathways

Toll-like receptors (TLRs) or nod-like receptors (NRLs) are pathogen recognition receptors of the innate immune system that recognize molecular patterns derived from microbes. [77] Fourteen studies assessed the effect of polymorphisms in 11 genes of the TLR and NLR signalling pathways on pneumococcal disease [29, 30, 33, 43, 50, 53, 57, 59, 63, 65, 67, 72, 74, 76]. Six polymorphisms were assessed in multiple studies and could be included in a meta-analysis. Five studies assessed the association between polymorphisms in *TLR2* (rs5743708) and *TLR4* (rs4986790) and susceptibility [30, 33, 50, 59, 74]. In the meta-analyses neither of the polymorphisms showed any effect. Rs352140 in *TLR9* was assessed in two studies for an association with susceptibility which resulted in no association in the separate studies and the meta-analysis [43, 74]. The *CD14* CC genotype of rs2569190 was significantly associated with susceptibility in a meta-analysis including two studies (OR 1·77, 95% CI 1·18–2·66) [33, 59]. Two studies including 224 patients and 284 controls studied rs4251513 of *IRAK4* and no effect was found on susceptibility in the meta-analysis [63, 65].

Polymorphisms in the Toll interleukin-1 receptor domain-containing adaptor protein (*TIRAP*) gene were investigated in three studies including five cohorts with in total 1601 white patients and 2826 African patients [29, 67, 76]. In the meta-analysis with the polymorphism rs8177374 was not associated with pneumococcal disease.

Three studies assessed the effect on outcome of polymorphisms in genes involved in pathogen recognition receptor signaling [50, 53, 65]. A Spanish study with 60 patients assessed the effect of 10 polymorphisms in *IRAK4, IRAK1, IRAKM* and *MYD88* on outcome of pneumococcal disease, but after re-calculation of their results the patients groups appeared too small to find significant assocations [65]. A study of 66 children with pneumococcal meningitis on the influence of *NOD1, NOD2, TLR2, TLR4, TLR9, TRAIL* and *CASP1* polymorphisms on susceptibility and outcome showed no significant associations after correction for multiple testing [53]^,^ [57].

#### Complement system

Mannose-binding lectin (MBL) is a soluble pattern recognition receptor of the collectin group that activates the lectin complement pathway after binding to a microorganism. Structural mutations in exon 1 of the *MBL2* gene resulting in variant allele B, C or D (rs1800450, rs1800451 or rs5030737), have been associated with reduced functional serum MBL levels [78].

**Fig. 2:**
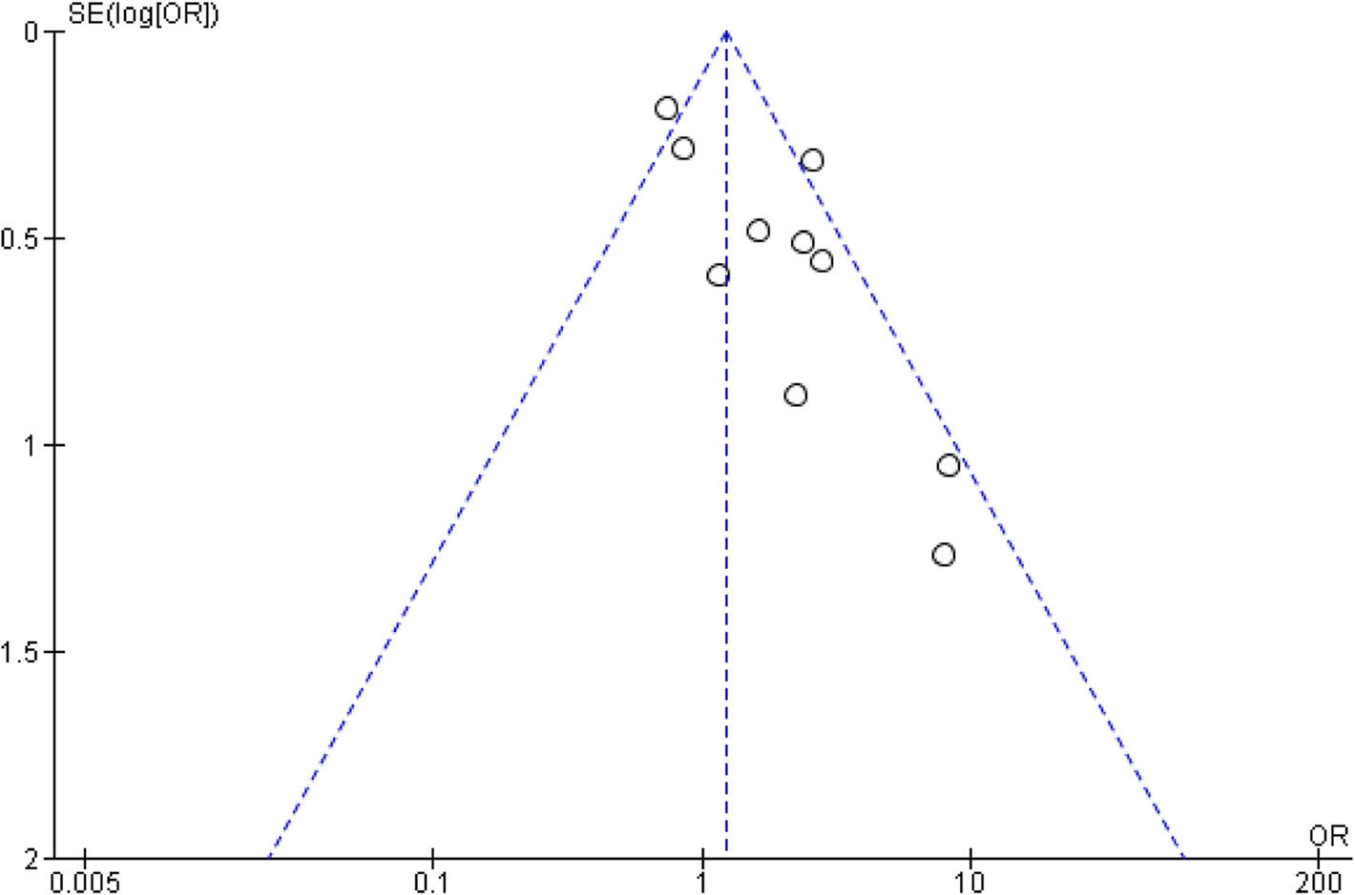
Funnel plot with *MBL2* studies. Funnel plot with studies assessing the effect of MBL2 variant allele B, C or D (rs1800450, rs1800451 or rs5030737) on pneumococcal disease susceptibility. Each dot represents one study. The vertical blue dashed line corresponds to the mean effect size on susceptibility. The outer dashed lines indicate the triangular region within which 95% of studies are expected to lie. SE: standard error as the measure of study size with a reversed scale (most powerful studies are placed towards the top), OR: odds ratio as the effect size of the studies on a log scale

The effect of *MBL2* variant allele B, C or D on susceptibility to pneumococcal disease was assessed in 9 studies which were included in the meta-analysis [18, 19, 27, 35, 49, 58, 62, 64, 76]. In the meta-analysis, 2504 patients and 4749 controls were included, and homozygosity of any of the variant alleles was significantly associated with susceptibility to pneumococcal disease (OR 1·67, 95% CI 1·04–2·69). A Funnel plot with the 10 study cohorts showed the overall effect on susceptibility was likely influenced by publication bias (Fig. 2). Effect on outcome of *MBL2* variant allele B, C or D was assessed in 10 studies, but only 3 of these studies could be included in the meta-analysis due to lacking of detailed genotypic data in the manuscripts [35, 58, 64]. The meta-analysis showed no significant effect on outcome of pneumococcal disease. Rs7096206 in the promotor region of *MBL2* was analysed in seven studies and yielded no significant association with susceptibility in the meta-analysis [18, 19, 27, 35, 49, 58, 62].

After binding of MBL to a pathogens surface, a serine protease called MASP (MBL-associated serine protease) is activated, which cleaves complement precursors to activated complement proteins further down the cascade [79]. Associations of polymorphisms in *MASP2* with pneumococcal disease were assessed in two studies, but showed no significant effect on outcome and susceptibility [34, 73].

Surfactant protein A or D (SFTPA, SFTPD) are also collectins and act as a first line of defence against microorganisms in the nasopharynx and respiratory tract by facilitating elimination of microorganisms [80]. A study of 7 *SFTPD* and *SFTPA* polymorphisms in 326 pneumococcal CAP patients and 1538 controls showed no association of these genes with susceptibility [44]. Another study of 182 European Americans (EA) and 53 African Americans (AA) with IPD assessed the effect on susceptibility of 24 polymorphisms in *SFTPA* and *SFTPD* [46]. Because genotypic data was not provided they could not be included in the meta-analysis. Their strongest associations were with two *SFTPD* polymorphisms (rs17886286 and rs12219080; OR 0.45, 95% CI 0.25–0.82and OR 0.32, 95% CI 0.13–0.78), not corrected for multiple testing [46].

L-Ficolin (encoded by *FCN2*) is a pattern-recognition molecule, that enhances phagocytosis and activates the lectin pathway of complement activation after binding to lipoteichoic acid or Gram-positive bacteria [81]. Five functional polymorphisms in *FCN2* were analysed in 290 patients with pneumococcal disease and in 720 controls yielding no associations with susceptibility [31].

After initiation of the three complement activation pathways the final common pathway is activated, in which C5 is converted into C5a,an important anaphylatoxin and a chemoattractant [82]. A Dutch study with 217 pneumococcal meningitis patients assessed the effect on outcome of 17 polymorphisms in 7 complement components further down the cascade [47]. This yielded 1 significant association of rs17611 in *C5* with unfavourable outcome (OR 2·25, 95% CI 1·33–3·81) after correction for multiple testing [47]. Another Dutch study investigated in the same population the effect of these complement components on susceptibility showing no significant associations after correction for multiple testing [56].

#### Fcγ receptors

Fc (fragment crystallizable) receptors are found on the surface of immune cells and bind to immunoglobulins (Ig). Of the 6 types of Fcγ receptors, FcγRIIa and FcγRIIIa exists as two allotypic variants with different binding affinity for IgG [83]. The more common F158 allotype of the *FCGR3A* gene has a lower IgG affinity than the V158 allotype (rs396991) [84]. For the *FCGR2A* gene the more common H131 allotype has a higher IgG affinity than the R131 allotype (rs1801274) [84]. Seven studies assessed the effect of rs1801274 (*FCGR2A*) on susceptibility and 3 assessed the effect on outcome of pneumococcal disease [17, 22, 24, 33, 37, 45, 50, 52]. The outcome studies lacked genotypic data for the meta-analysis and one study on susceptibility was excluded, because patient overlap with another study [22, 33]. In the meta-analysis on susceptibility 6 studies with a total of 570 patients and 4972 controls were included and no overall effect was found [17, 24, 33, 37, 45, 52]. One study assessed the effect of rs396991 (*FCGR3A*) in 85 bacteraemia pneumococcal pneumonia patients and 1224 healthy controls, showing no effect on susceptibility and outcome [45].

#### NFκβ signalling pathway

NFκB (nuclear factor kappa-light-chain-enhancer of activated B cells) is a transcriptional regulator important for both the adaptive and innate immune response [85]. Six studies investigated the effect of polymorphisms in genes coding for modulators of the NFκB signalling pathway on outcome and susceptibility of pneumococcal disease [32, 38, 40, 55, 67, 75]. Five polymorphisms in genes coding for NFκB inhibitors could be analysed in a meta-analysis. The effect of polymorphisms in *NFKBIA* and *NFKBIE* (rs3138053, rs2233406, rs529948) on susceptibility was assessed in two studies, revealing no significant associations in the meta-analyses [32, 67]. Two other polymorphisms in the NFκB inhibitor genes *NFKBIZ* (rs616597) and *NFKBIL2* (rs760477) were assessed in 3 cohorts for an effect on susceptibility and meta-analysis showed no significant associations [38, 40, 67]. A study including 531 adult pneumococcal meningitis patients and 376 controls studied two polymorphisms in *CARD8* and *NLRP1* both coding for proteins required for activation of NFκB or caspases in the context for inflammation or apoptosis respectively [85]. This study showed an association of rs2043211 in *CARD8* with poor outcome (OR 2·10, 95% CI 1·04–4·21) and rs11651270 in *NLRP1* with death (OR 2·32, 95% CI 1·12–4·78), but this was not significant after correction for multiple testing [55].

#### Cytokines

Cytokines are important molecules mediating cell signalling and include small proteins like chemokines, interferons, interleukins (ILs), lymphokines, or tumor necrosis factors (TNFs) [86, 87] Seven studies assessed the effect of polymorphisms in 11 cytokine genes on susceptibility, disease phenotype and outcome of pneumococcal disease [21, 23, 41, 42, 48, 68, 75]. The polymorphism rs1800795 in *IL6* was assessed in two studies, showing no effect on susceptibility in the meta-analysis [23, 48]. One Spanish study with 144 IPD patients and 280 controls assessed the effect on susceptibility of 33 polymorphisms in the genes coding for IL-10, IL-12B, IL-1A, IL-1B, IL-R1 and IL-4 [75]. None were significantly associated after correction for multiple testing [75].

Macrophage migrating inhibitory factor (MIF) is a pro-inflammatory cytokine acting at the interface of the immune and endocrine systems [88]. The effect of polymorphisms in *MIF* on pneumococcal disease were investigated in one phenotype study showing effect of the high expression allele (rs5844572) on developing the meningitis phenotype and one outcome study showing effect of high expression alleles (rs5844572, rs755622) on unfavourable outcome and death [41, 68].

#### Coagulation and fibrinolysis factors

During severe infection the inflammatory response shifts the haemostatic balance towards a pro-coagulant state, which can lead to diffuse intravascular coagulation and organ damage [89]. Three studies assessed the effect of polymorphisms in coagulation or fibrinolysis genes on susceptibility and outcome of pneumococcal disease [39, 60, 66]. A study investigated the effect of the factor V Leiden (FVL) mutation (rs6025) in 163 patients and 8147 controls on IPD susceptibility and outcome, showing no significant associations [39].

Carboxypeptidase B2 (*CPB2),* also known as thrombin-activatable fibrinolysis inhibitor (TAFI), plays an anti-fibrinolytic role during fibrin clot degradation and an anti-inflammatory role by inactivating pro-inflammatory mediators, such as complement activation products [90]. A study with 716 pneumococcal meningitis patients studied the effect of polymorphisms in carboxypeptidase B2 (*CPB2*, rs1926447, rs3742264) on disease susceptibility and outcome [66]. No effect was found on susceptibility, but rs3742264 was associated with developing systemic complications (OR 0·40, 95% CI 0·20–0·79) [66].

Plasminogen activator inhibitor 1 (PAI-1) inhibits the pro-fibrinolytic enzymes urokinase and tissue plasminogen activator and thereby modulates fibrinolysis [91]. The effect of rs1799889 in the gene coding for PAI-1 (*SERPINE1*) on pneumococcal meningitis outcome was studied in a Dutch study with 400 patients and they found an effect on occurrence of cerebral infarction (OR 2·20, 95% CI 1·19–4·07), unfavourable outcome (OR 1·69, 95% CI 1·03–2·78) and mortality (OR 2·20, 95% CI 1·02–4·86) [60].

#### Other factors

Eight studies focused on genes that could not be categorized in the other subcategories. Two of these studies assessed the role of polymorphisms in the gene coding for C-reactive protein (CRP) in pneumococcal disease. *CRP* contains a dinucleotide repeat polymorphism in the intron region (rs3138528) which was assessed in a study with 205 IPD patients and 345 controls, showing significantly more patients had the 134 base pair allele than controls (OR 1·52, 95% CI 1·18–1·96) [20]. Another study investigated the effect of 3 polymorphisms in *CRP* (rs1800947, rs2794521, rs1130864) on outcome in 42 patients with a pneumococcal bacteraemia and found an association with mortality and rs2794521 (OR 9·6, 95% CI 1·3–72·5), not corrected for multiple testing [28].

Protein thyrosine phosphatases (PTPs) regulate the immune response through influencing the responsiveness of B and T cell receptors [92]. Rs2476601in the gene coding for PTP non-receptor type 22 (*PTPN22*) was assessed in two studies with in total 1492 IPD patients and 2050 controls [25, 67]. The meta-analysis showed no effect on susceptibility [25].

Nitric oxide synthase 2 (NOS2) is an enzyme encoded by the *NOS2* gene, which is involved in nitric oxide production and apoptosis of macrophages [93]. Nine polymorphisms in *NOS2* were investigated in a Malawian study, showing no influence of any of the variants on IPD susceptibility or survival [36].

One study investigated if rs37972 in the glucocorticoid-induced transcript 1 gene (*GLCCI1*) influenced disease outcome and the response to glucocorticosteroids in pneumococcal meningitis [54]. The function of *GLCCI1* unknown, but it is expressed in both lung cells and immune cells and may be an early marker of glucocorticoid-induced apoptosis [94]. No association was found between rs37972 and mortality rates per dexamethasone treatment group [54].

Studies have showed bacteria are able to hijack the β2-adrenoceptor and thereby stabilize its binding to the endothelium which could enhance crossing the blood-brain barrier [95]. The effect of 2 functional polymorphisms in the β2-adrenoceptor (*ADRB2)* gene on susceptibility and outcome of pneumococcal meningitis was studied in 396 patients and 376 controls [51]. Rs1042714 of *ADRB2* was associated with susceptibility (OR 1·52, 95% CI 1·12–2·07) but had no influence on outcome of disease [51].

#### Studies with hypothesis free approach

Five studies had a hypothesis free approach to find (new) genetic variations associated with pneumococcal disease. Two of them were sequencing studies in a selected group of genes [63, 72]. The first study sequenced 3 genes involved in the Toll-like receptor signalling pathway: *MYD88, IRAK4, IKBKG* (inhibitor of nuclear factor kappa-B kinase subunit gamma) of 164 IPD patients and 164 controls [63]. After sequencing 233 variants were identified of which one (rs4251545 in *IRAK4*) had a minor allele frequency (MAF) of more than 5%. This variant was associated with susceptibility to IPD (OR 1·50; 95% CI 1·10–2·04; *p* = 9·96 × 10^− 3^) but after correction for multiple testing this polymorphism did not retain statistical significance [63].

The other sequencing study sequenced 46 innate immune genes of 435 patients and 416 controls to assess the influence on outcome and susceptibility to pneumococcal meningitis [72]. They identified 2099 variations of which 80% had a MAF below 1% (1854 variations for susceptibility and 1385 for outcome). Neither the single nucleotide polymorphism (SNP) or haplotype analysis nor the analysis for association between a set of rare variants and phenotypes, reached the significance level after correction for multiple testing. The strongest associations with susceptibility were in *CARD8*, rs2008521 (OR 1·82; CI 1·28–2·75; *p* = 8·2 × 10^− 4^) and in *CXCL1*, rs56078309 (OR 1·96; CI 1·34–2·87; *p =* 8·2 × 10^− 4^) and with outcome were in *IRAK4*, rs4251552 (OR 2.86; CI 1·58–5·18; *p* = 4·8 × 10^− 4^) and *NOD2*, rs2067085 (OR 2·16; CI 1·40–3·34; *p* = 5·1 × 10^− 4^) [72].

Two of the hypothesis free studies were exome wide association studies performed in the same Dutch cohort of pneumococcal meningitis patients [69, 71]. Genotyping of subjects in these studies was done with an Illumina BeadChip consisting of more than 240,000 markers, with approximately 75% of these markers having a MAF below 5%. The first study assessed susceptibility to pneumococcal meningitis and included 469 patients and 2072 controls and a total of 100,464 polymorphisms passed quality control thresholds [71]. The strongest associations with susceptibility were rs139064549 in *COL11A1* (OR 3·21; 95% CI 2·05–5·02; *p* = 1·51 × 10^− 6^) and rs9309464 in *EXOC6B* (OR 0·66; 95% CI 0·54–0·81; *p* = 6·01 × 10^− 5^), both did not reach the exome wide significance level [71]. The study on outcome included 472 culture proven pneumococcal meningitis patients and their strongest association was in *AKT3*, rs10157763 (OR 1·88; 95% CI 1·4–2·6; *p* = 9·9 × 10^− 5^) but this was not significant after correction for multiple testing [69].

The fifth hypothesis free study was a genome wide association study on pneumococcal bacteraemia susceptibility in 429 Kenyan children and 2677 controls [70]. In this study samples were genotyped with an Affymetrix® SNP chip and polymorphisms not passing the quality control with a MAF of less than 1%, a HWE of *p* < 1 × 10^− 20^ and a missingness of more than 2%, were excluded for imputation. After sample and SNP quality control 787,861 genotyped autosomal SNPs were left for analysis, which were extended to 10,996,499 autosomal SNPs after imputation. The study identified an association which reached the genome wide significance threshold between rs140817150in a long intergenic non-coding RNA (lincRNA) gene (*AC011288.2*) and pneumococcal bacteraemia susceptibility and replicated the results in a replication cohort with 113 children and 1136 controls (OR 2·47, 95% CI 1·84–3·31, p-combined = 1·69 × 10^− 9^) [70].

## Discussion

We identified 60 studies evaluating host genetic variations in 16,034 patients with pneumococcal disease. Meta-analyses showed that genetic variants in the genes *CD14* (rs2569190) and *MBL2* (one of the variant alleles rs1800450, rs1800451 or rs5030737) were associated with susceptibility to pneumococcal disease*.* A hypothesis free approach was applied in few studies resulting in one genome wide significant association in a gene coding for lincRNA (rs140817150) with IPD susceptibility which was replicated in an independent IPD cohort.

Few findings were replicated in independent cohorts. Replication generally led to negative results, or – in case of *MBL2* – careful analysis suggested considerable publication bias. The role of genetic variation on outcome was evaluated in about half of identified studies, but results were not confirmed because of the lack of detailed clinical metadata and heterogeneity of definitions and outcomes. To ease replication, international collaboration between study groups on genetics in pneumococcal disease is needed to ensure uniform research designs and outcome measures [96, 97]. This should lead to an open source research register for genetic associations studies, evaluating host and pathogen genetic data of pneumococcal disease, to facilitate data exchange and prevent publication bias. Such team-science effort is needed to decrease methodological flaws and contribute to more robust findings on the genetic basis of pneumococcal disease, a disease with enormous impact on global health [1, 97].

The significantly associated polymorphisms in the meta-analysis, in *CD14* (rs2569190) and *MBL2* (one of the variant alleles of rs1800450, rs1800451 or rs5030737) are known functional polymorphisms. The variant alleles of *MBL2* have structural differences which are associated with decreased MBL concentrations and thereby decreased activation of the complement system [98]. Soluble CD14 (sCD14) is a pattern recognition receptor and acts as a co-receptor of TLR-4 to bind microbial components to endothelial and epithelial cells [99]. The risk allele T of rs2569190 for pneumococcal disease susceptibility in our meta-analysis, is associated with high sCD14 levels in expression studies [100, 101]. Our findings correspond with other studies showing the T allele is associated with an increased occurrence of sepsis and increased serum sCD14 levels in patients with risk genotypes [102] [103]. Although the causal allele might be not the association signal due to linkage disequilibration, these studies are suggestive for a causal relationship of genetic variation in both *MBL2* or *CD14* and susceptibility to pneumococcal disease.

The results of our meta-analyses should be interpreted with caution because many included methodologically flawed studies. First of all, sample sizes were often inadequate, whereby robust conclusions on the influence of the studied genetic variants could not be drawn. In studies focusing on outcome, small sample sizes result in few unfavourable events per study group and consequentially limited study power. Second, in most studies data collection was retrospective which might have led to missing data. Many studies had a retrospective inclusion design which poses a risk for to selection bias as reflected by the extremely low mortality rates among included patients. In other studies DNA was not available for a considerable proportion of patients, particularly those with more severe disease passing away before DNA collection. Inclusion of patients with less severe disease decreases study power and could underestimate influence of polymorphisms on severity or mortality of pneumococcal disease. Third, case selection differed between studies. Different phenotypes of pneumococcal disease, ethnicities and age categories were studied which could possibly limit the meta-analysis. In 30% of the studies ethnicity was mixed or not specified, which could be a major source for bias since frequencies of polymorphic genetic loci vary substantially between ethnic groups. Furthermore, control populations were heterogeneously selected and only 8 cohorts (of 57 cohorts; 14%) matched for both age and sex. Fourth, quality control procedures for DNA extraction and genotyping were rarely specified. Only half of the studies which determined genotypes by PCR followed by allelic discrimination methods (21 of 41 studies) stated they confirmed genotypes by sequencing or retesting of samples. In the candidate gene studies only 15 (27%) described the genotyping success rate and 7 (13%) blinding of laboratory personal. Four out of the five hypothesis free studies described extensive quality control procedures like genotyping accuracy, calling rates, and rates of missing samples [69–72]. Finally, statistical analyses differed between studies leading to different effect sizes or different cut-offs for significant associations. Logistic regression with correction for confounders was done in only half of the studies and about one third of the studies that assessed three or more polymorphisms did not correct for multiple testing.

In recent years, many loci have been identified by GWAS, since the cost of genotyping SNPs decreased and the cohort sizes increased [104]. Despite the success in identifying disease loci, understanding of how polymorphisms predispose individuals to disease remains limited [104]. Besides methodological flaws, it is likely single genes or genetic variants do not control susceptibility and outcome of complex traits. Probably most heritability can be explained by effects on genes outside core pathways due to interconnection with genes in regulatory networks expressed in disease-relevant cells [105]. In order to understand the genetics of complex traits future studies should focus on gene-gene interactions as well [97]. Other future approaches for increasing our understanding in heritability could be targeted or whole-genome sequencing in people with extreme phenotypes, in order to find variants in the lower frequency with larger effect domains [97]. Besides reference panels of genomic variation should be adequately used to enhance coverage of existing and future GWAS and methods for detection of copy number variants and other structural variants could be improved [97]. Besides all this, functional understanding of these variants is needed for better insight in pathogenesis of disease and drug discovery. For example the whole genome association study of the Kenyan Bacteraemia Study Group explored the functionality of a polymorphism in a gene coding for lincRNA, with a qPCR to quantify levels of RNA expression in leukocyte cell subtypes, observing elevation only in neutrophils [70]. Most of the studies included in this review investigated a functional role of identified polymorphisms by measuring serum of CSF protein expression, [20, 26, 28, 34, 41, 42, 44, 47, 55, 56, 58, 60, 66, 69, 72] but not all were able to demonstrate a functional effect. Moreover the majority of the studies (70%) did not analyse the functionality of the genetic variants.

## Conclusions

Several host genetic polymorphisms have been identified to influence susceptibility and outcome of pneumococcal disease, but most of these studies are hampered by methodological flaws or were not reproduced (yet). Carefully designed whole-genome association and replication studies are needed with detailed clinical meta-data to further clarify and confirm the genetic basis of pneumococcal disease. To improve our understanding in the functionality of polymorphisms the next step is to investigate the downstream molecular effects of polymorphisms with large-scale clinical cohort studies within a specific acute illness as pneumococcal disease.

## Additional files

Additional file 1: **Table S1.** Synonyms of genetic variants (DOCX 13 kb)

Additional file 2: Meta-analyses of genetic association studies on susceptibility and outcome of pneumococcal disease. (PDF 505 kb)

## Data Availability

The datasets used and/or analysed during the current study are available from the corresponding author on reasonable request.

## Abbreviations

AA: African Americans
ADRB2: β2-adrenoceptor
CAP: Community acquired pneumococcal pneumonia
CPB2: Carboxypeptidase B2
CRP: C-reactive protein
EA: European Americans
Fc: Fragment crystallizable
FVL: Factor V Leiden
GLCCI1: Glucocorticoid-induced transcript 1 gene
Ig: Immunoglobulin
IKBKG: Inhibitor of nuclear factor kappa-B kinase subunit gamma
IL: Interleukin
IPD: Invasive pneumococcal disease
lincRNA: Long intergenic non-coding RNA
MAF: Minor allele frequency
MASP: MBL-associated serine protease
MBL: Mannose-binding lectin
MIF: Macrophage migrating inhibitory factor
NFκB: Nuclear factor kappa-light-chain-enhancer of activated B cells
NLR: Nod-like receptor
NOS2: Nitric oxide synthase 2
PAI-1: Plasminogen activator inhibitor 1
PCR: Polymerase chain reaction
PTP: Protein thyrosine phosphatase
SFTPA: Surfactant protein A
SFTPD: Surfactant protein D
SNP: Single nucleotide polymorphism
TAFI: Thrombin-activatable fibrinolysis inhibitor
TIRAP: Toll interleukin-1 receptor domain-containing adaptor protein
TLR: Toll-like receptor
TNF: Tumor necrosis factor
