# Supplementary material for: Host genetic variability and pneumococcal disease: a systematic review and meta-analysis
Source: BMC Med Genomics. 2019 Sep 13;12:130. doi: 10.1186/s12920-019-0572-x (PMC6743160; doi:10.1186/s12920-019-0572-x)
Supplement: Supplementary file 1 — Table S1. Synonyms of genetic variants (DOCX 13 kb) [file 12920_2019_572_MOESM1_ESM.docx]

**Additional file 1: Table S1 - synonyms of genetic variants**

| **Gene** | **Rs number** | **Synonyms** |
| --- | --- | --- |
| *MBL2* | rs11003125 | -550 H/L (-551 in article), G/C |
| *FCN2* | rs17514136 | -4A>G |
| *FCN2* | rs17549193 | Thr236Met |
| *MBL2* | rs1800450 | allele B, codon 54, +54, G/A |
| *MBL2* | rs1800451 | allele C, codon 57, +57, G/A |
| *TNF* | rs1800629 | -308G/A |
| *IL6* | rs1800795 | -174 G/C |
| *IL10* | rs1800896 | -G1082A |
| *FCGR2A* | rs1801274 | H131R, A/G |
| *NOD2* | rs2066844 | +2209 A>T |
| *NOD2* | rs2066845 | 2722 G>T |
| *CASP1* | rs2066847 | +3020 insC |
| *CASP1* | rs2282659 | B43 A>G |
| *PTPN22* | rs2476601 | 1858C>T, R620W |
| *CD14* | rs2569190 | -159 C/T |
| *FCN2* | rs3124952 | -986G>A |
| *FCN2* | rs3124953 | -602G>A |
| *CRP* | rs3138528 | dinucleotide repeats |
| *TLR9* | rs352140 | +2848 G>A |
| *TLR4* | rs4986790 | D299G, -Asp/Gly299, +896 A>G |
| *TLR4* | rs4986791 | -Thr/Ile399 |
| *MBL2* | rs5030737 | Allele D, codon 52, +52, C/T |
| *TLR2* | rs5743703 | R579H |
| *TLR2* | rs5743704 | P631H |
| *TLR2* | rs5743708 | -Arg/Gln753, R753Q, +2477 G>A |
| *TLR9* | rs5743836 | -1237 T>C |
| *MIF* | rs5844572 | CATT7 |
| *FVL* | rs6025 | Arg506Gln |
| *NOD1* | rs6958571 | +32656 C>A |
| *MBL2* | rs7095891 | +4 P/Q, C/T |
| *MBL2* | rs7096206 | -221 (G>C) or (Y>X) |
| *MASP2* | rs72550870 | D105G |
| *MIF* | rs755622 | −173 G/C |
| *FCN2* | rs7851696 | Ala258Ser |
| *TIRAP* | rs8177374 | S180L |
| *LTA* | rs909253 | +252A/G |
