# Supplementary material for: Host genetic variability and pneumococcal disease: a systematic review and meta-analysis
Source: BMC Med Genomics. 2019 Sep 13;12:130. doi: 10.1186/s12920-019-0572-x (PMC6743160; doi:10.1186/s12920-019-0572-x)
Supplement: Supplementary file 2 — Meta-analyses of genetic association studies on susceptibility and outcome of pneumococcal disease. (PDF 505 kb) [file 12920_2019_572_MOESM2_ESM.pdf]

**Additional file 2 - Meta-analyses of genetic association studies on susceptibility and outcome of pneumococcal disease**

***a. Susceptibility studies †***

Forest plot a.1 *CD14* – CC genotype of rs2569190*

| Study or Subgroup | Patients Events | Patients Total | Control Events | Control Total | Odds Ratio M-H, Fixed, 95% CI | Year |
|---|---|---|---|---|---|---|
| Yuan, 2008 | 30 | 85 | 100 | 409 | 1.69 [1.02, 2.78] | 2008 |
| Tellería-Orriols, 2014 | 39 | 114 | 14 | 66 | 1.93 [0.95, 3.91] | 2014 |
| **Total (95% CI)** | | **199** | | **475** | **1.77 [1.18, 2.66]** | |
| Total events | 69 | | 114 | | | |

Heterogeneity: Chi² = 0.10, df = 1 (P = 0.76); I² = 0%
Test for overall effect: Z = 2.75 (P = 0.006)

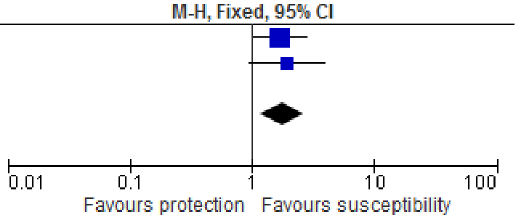


* Human Genome Variation Society (HGVS) representation: NM_000591.3(CD14):c.-260T>C

Forest plot a.2 *FCGR2A* – GG genotype (R131/R131) of rs1801274*

| Study or Subgroup | Patients Events | Patients Total | Control Events | Control Total | Odds Ratio M-H, Random, 95% CI | Year |
|---|---|---|---|---|---|---|
| Yee, 2000 | 21 | 42 | 40 | 136 | 2.40 [1.18, 4.87] | 2000 |
| Moens, 2006 | 12 | 55 | 31 | 100 | 0.62 [0.29, 1.34] | 2006 |
| Yuan, 2008 | 33 | 85 | 84 | 409 | 2.46 [1.49, 4.04] | 2008 |
| Endeman, 2009 | 17 | 73 | 77 | 313 | 0.93 [0.51, 1.70] | 2009 |
| Solé-Violán, 2011 | 18 | 85 | 310 | 1224 | 0.79 [0.46, 1.35] | 2011 |
| Bouglé, 2012 | 53 | 243 | 697 | 2789 | 0.84 [0.61, 1.15] | 2012 |
| **Total (95% CI)** | | **583** | | **4971** | **1.14 [0.73, 1.80]** | |
| Total events | 154 | | 1239 | | | |

Heterogeneity: Tau² = 0.24; Chi² = 21.38, df = 5 (P = 0.0007); I² = 77%
Test for overall effect: Z = 0.58 (P = 0.56)

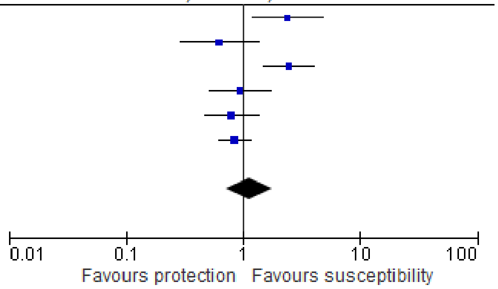


* HGVS representation: NM_021642.3(FCGR2A):c.497A>G (p.His166Arg)

Forest plot a.3 *IL6* – GG genotype of rs1800795*

| Study or Subgroup | Patients Events | Patients Total | Controls Events | Controls Total | Odds Ratio M-H, Fixed, 95% CI | Year |
|---|---|---|---|---|---|---|
| Schaaf, 2005 | 29 | 100 | 17 | 50 | 0.79 [0.38, 1.64] | 2005 |
| Martin-Loeches, 2012 | 136 | 306 | 581 | 1227 | 0.89 [0.69, 1.14] | 2012 |
| **Total (95% CI)** | | **406** | | **1277** | **0.88 [0.69, 1.11]** | |
| Total events | 165 | | 598 | | | |

Heterogeneity: Chi² = 0.09, df = 1 (P = 0.77); I² = 0%
Test for overall effect: Z = 1.06 (P = 0.29)

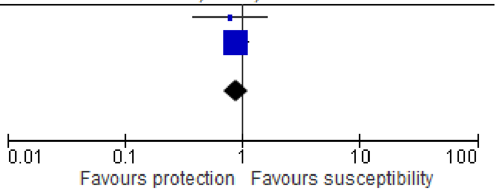


* HGVS representation: NC_000007.14(IL6):g.22727026C>G

Forest plot a.4 *IRAK4* – G allele of rs4251513*

| Study or Subgroup | Patients Events | Patients Total | Control Events | Control Total | Odds Ratio M-H, Random, 95% CI | Year |
|---|---|---|---|---|---|---|
| Ellis, 2015 | 178 | 328 | 145 | 328 | 1.50 [1.10, 2.04] | 2015 |
| Carrasco-Colom, 2015 | 44 | 110 | 121 | 236 | 0.63 [0.40, 1.00] | 2015 |
| **Total (95% CI)** | | **438** | | **564** | **0.99 [0.43, 2.30]** | |
| Total events | 222 | | 266 | | | |

Heterogeneity: Tau² = 0.33; Chi² = 9.31, df = 1 (P = 0.002); I² = 89%
Test for overall effect: Z = 0.02 (P = 0.98)

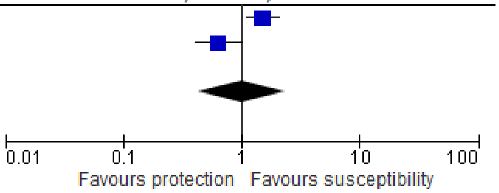


* HGVS representation: NC_000012.12(IRAK4):g.43780401G>C

Forest plot a.5 *MBL2* – variant alleles (O/O genotype) of rs1800450, rs1800451 or rs5030737 *

| Study or Subgroup | Patients Events | Patients Total | Controls Events | Controls Total | Odds Ratio M-H, Random, 95% CI | Year |
|---|---|---|---|---|---|---|
| Kronborg, 2002 | 9 | 140 | 7 | 250 | 2.38 [0.87, 6.55] | 2002 |
| Roy, 2002 | 28 | 229 | 18 | 353 | 2.59 [1.40, 4.81] | 2002 |
| Moens, 2006 | 7 | 63 | 7 | 162 | 2.77 [0.93, 8.24] | 2006 |
| Endeman, 2008 | 4 | 60 | 13 | 223 | 1.15 [0.36, 3.68] | 2008 |
| Garcia-Loarden, 2012 cohort 1 | 15 | 340 | 89 | 1736 | 0.85 [0.49, 1.50] | 2012 |
| Garcia-Loarden, 2012 cohort 2 | 4 | 84 | 2 | 91 | 2.23 [0.40, 12.48] | 2012 |
| Brouwer, 2013 | 11 | 299 | 1 | 216 | 8.21 [1.05, 64.09] | 2013 |
| Lundbo, 2014 | 52 | 1180 | 70 | 1192 | 0.74 [0.51, 1.07] | 2014 |
| Mills, 2015 | 6 | 95 | 19 | 477 | 1.63 [0.63, 4.18] | 2015 |
| Gowin, 2018 | 2 | 14 | 1 | 49 | 8.00 [0.67, 95.76] | 2018 |
| **Total (95% CI)** | | **2504** | | **4749** | **1.67 [1.04, 2.69]** | |
| Total events | 138 | | 227 | | | |

Heterogeneity: Tau² = 0.30; Chi² = 24.61, df = 9 (P = 0.003); I² = 63%
Test for overall effect: Z = 2.11 (P = 0.03)

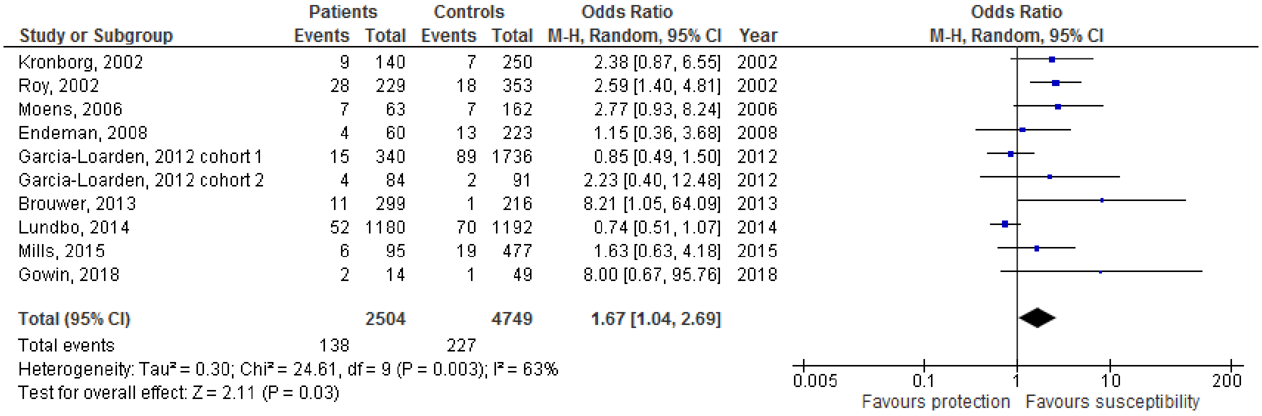


* HGVS representations: rs1800450; (NM_000242.2(MBL2):c.161G>A (p.Gly54Asp). rs1800451; NM_000242.2(MBL2):c.170G>A (p.Gly57Glu); rs5030737; NM_000242.2(MBL2):c.154C>T (p.Arg52Cys)

Forest plot a.6 *MBL2* – variant allele (X/X genotype) of rs7096206*

| Study or Subgroup | Patients Events | Patients Total | Controls Events | Controls Total | Odds Ratio M-H, Fixed, 95% CI | Year |
|---|---|---|---|---|---|---|
| Roy, 2002 | 9 | 229 | 14 | 353 | 0.99 [0.42, 2.33] | 2002 |
| Kronborg, 2002 | 6 | 140 | 12 | 250 | 0.89 [0.33, 2.42] | 2002 |
| Moens, 2006 | 3 | 63 | 3 | 162 | 2.65 [0.52, 13.49] | 2006 |
| Endeman, 2008 | 6 | 60 | 12 | 223 | 1.95 [0.70, 5.44] | 2008 |
| Garcia-Loarden, 2012 cohort 1 | 15 | 340 | 62 | 1736 | 1.25 [0.70, 2.22] | 2012 |
| Brouwer, 2013 | 17 | 299 | 11 | 215 | 1.12 [0.51, 2.44] | 2013 |
| Lundbo, 2014 | 49 | 1180 | 52 | 1192 | 0.95 [0.64, 1.42] | 2014 |
| **Total (95% CI)** | | **2311** | | **4131** | **1.09 [0.84, 1.42]** | |
| Total events | 105 | | 166 | | | |

Heterogeneity: Chi² = 3.27, df = 6 (P = 0.77); I² = 0%
Test for overall effect: Z = 0.65 (P = 0.51)

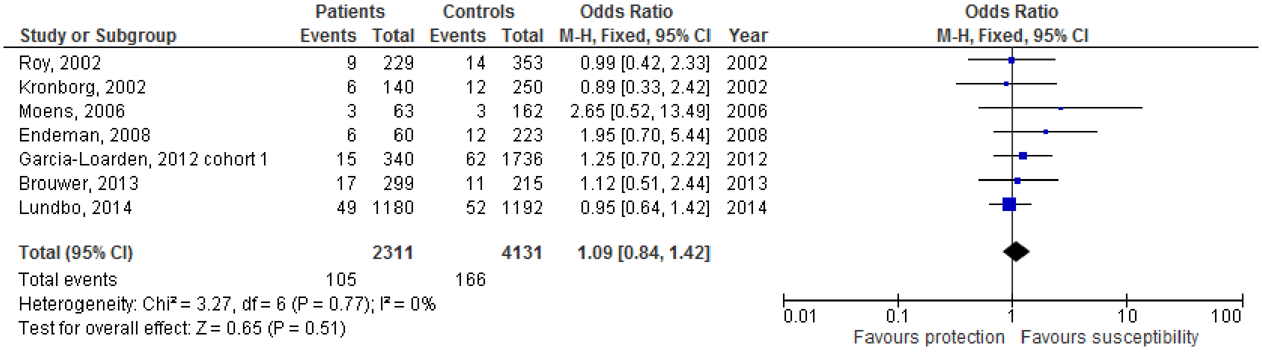


* HGVS representation: NC_000010.11(MBL2):g.52771925G>A,

Forest plot a.7 *NFKBIA* - BB+AB genotype (carriers variant allele) of rs3138053*

| Study or Subgroup | Patients Events | Patients Total | Controls Events | Controls Total | Odds Ratio M-H, Random, 95% CI | Year |
|---|---|---|---|---|---|---|
| Chapman, 2007 | 91 | 226 | 392 | 766 | 0.64 [0.48, 0.87] | 2007 |
| Lundbo, 2015 | 591 | 1193 | 588 | 1239 | 1.09 [0.93, 1.27] | 2015 |
| **Total (95% CI)** | | **1419** | | **2005** | **0.85 [0.51, 1.42]** | |
| Total events | 682 | | 980 | | | |

Heterogeneity: Tau² = 0.12; Chi² = 9.12, df = 1 (P = 0.003); I² = 89%
Test for overall effect: Z = 0.62 (P = 0.53)

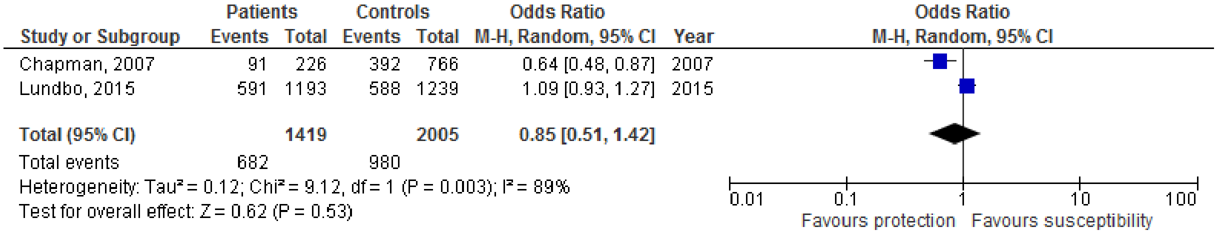


* HGVS representation: NC_000014.9(NFKBIA):g.35405648T>C

Forest plot a.8 *NFKBIA* - BB+AB genotype (carriers variant allele) of rs2233406*

| Study or Subgroup | Patients Events | Patients Total | Controls Events | Controls Total | Odds Ratio M-H, Random, 95% CI | Year |
|---|---|---|---|---|---|---|
| Chapman, 2007 | 96 | 260 | 397 | 762 | 0.54 [0.40, 0.72] | 2007 |
| Lundbo, 2015 | 585 | 1198 | 594 | 1245 | 1.05 [0.89, 1.23] | 2015 |
| **Total (95% CI)** | | **1458** | | **2007** | **0.76 [0.40, 1.45]** | |
| Total events | 681 | | 991 | | | |

Heterogeneity: Tau² = 0.21; Chi² = 15.58, df = 1 (P < 0.0001); I² = 94%
Test for overall effect: Z = 0.83 (P = 0.41)

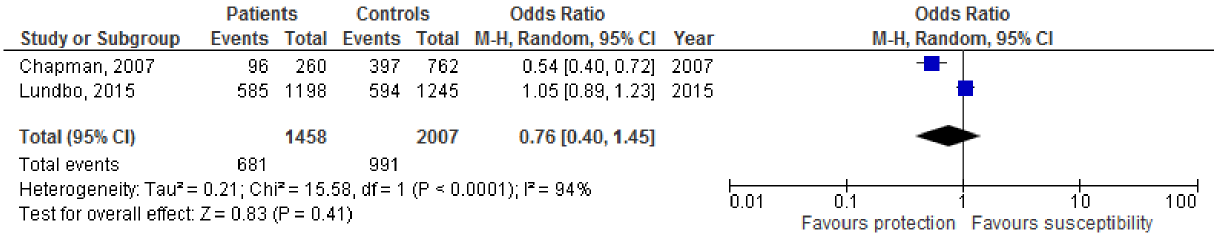


* HGVS representation: NC_000014.9(NFKBIA):g.35405593G>A

Forest plot a.9 *NFKBIE* - BB+AB genotypes (carriers variant allele) of rs529948*

| Study or Subgroup | Patients Events | Patients Total | Controls Events | Controls Total | Odds Ratio M-H, Random, 95% CI | Year |
|---|---|---|---|---|---|---|
| Chapman, 2007 | 57 | 288 | 222 | 756 | 0.59 [0.43, 0.83] | 2007 |
| Lundbo, 2015 | 312 | 1203 | 250 | 1246 | 1.40 [1.15, 1.69] | 2015 |
| **Total (95% CI)** | | **1491** | | **2002** | **0.92 [0.40, 2.13]** | |
| Total events | 369 | | 472 | | | |

Heterogeneity: Tau² = 0.35; Chi² = 19.46, df = 1 (P < 0.0001); I² = 95%
Test for overall effect: Z = 0.19 (P = 0.85)

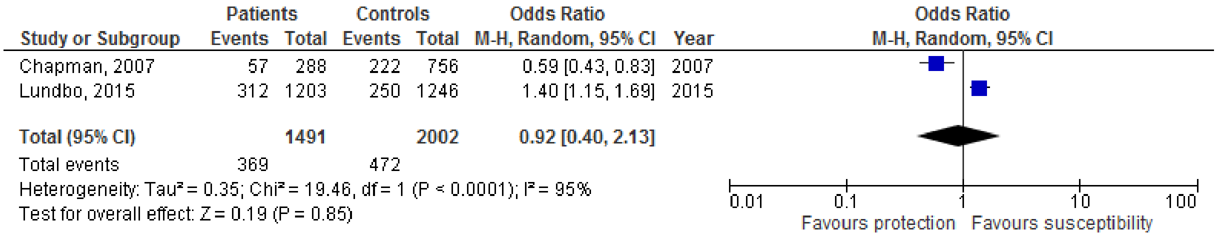


* HGVS representation: NC_000006.12(NFKBIE):g.44268632A>G

Forest plot a.10 *NFKBIL2* - AA genotype (wildtype) of rs760477*

| Study or Subgroup | Patients Events | Patients Total | Controls Events | Controls Total | Odds Ratio M-H, Fixed, 95% CI | Year |
|---|---|---|---|---|---|---|
| Chapman, 2010 cohort 2 | 93 | 165 | 255 | 550 | 1.49 [1.05, 2.12] | 2010 |
| Chapman, 2010 cohort 1 | 82 | 262 | 188 | 716 | 1.28 [0.94, 1.74] | 2010 |
| Lundbo, 2015 | 363 | 1183 | 358 | 1201 | 1.04 [0.88, 1.24] | 2015 |
| **Total (95% CI)** | | **1610** | | **2467** | **1.15 [1.00, 1.32]** | |
| Total events | 538 | | 801 | | | |

Heterogeneity: Chi² = 3.82, df = 2 (P = 0.15); I² = 48%
Test for overall effect: Z = 1.97 (P = 0.05)

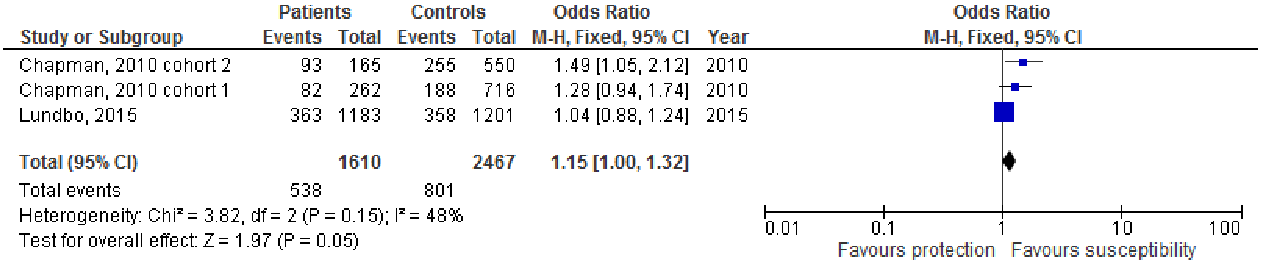


* HGVS representation: NC_000008.11(NFKBIL2):g.144443060G>A,

Forest plot a.11 *NFKBIZ* - BB+AB genotypes (carriers variant allele) of rs616597*

| Study or Subgroup | Patients Events | Patients Total | Controls Events | Controls Total | Odds Ratio M-H, Fixed, 95% CI | Year |
|---|---|---|---|---|---|---|
| Chapman, 2010 cohort 2 | 10 | 169 | 56 | 524 | 0.53 [0.26, 1.05] | 2010 |
| Chapman, 2010 cohort 1 | 96 | 265 | 282 | 705 | 0.85 [0.64, 1.14] | 2010 |
| Lundbo, 2015 | 472 | 1212 | 513 | 1247 | 0.91 [0.78, 1.07] | 2015 |
| **Total (95% CI)** | | **1646** | | **2476** | **0.88 [0.76, 1.01]** | |
| Total events | 578 | | 851 | | | |

Heterogeneity: Chi² = 2.35, df = 2 (P = 0.31); I² = 15%
Test for overall effect: Z = 1.88 (P = 0.06)

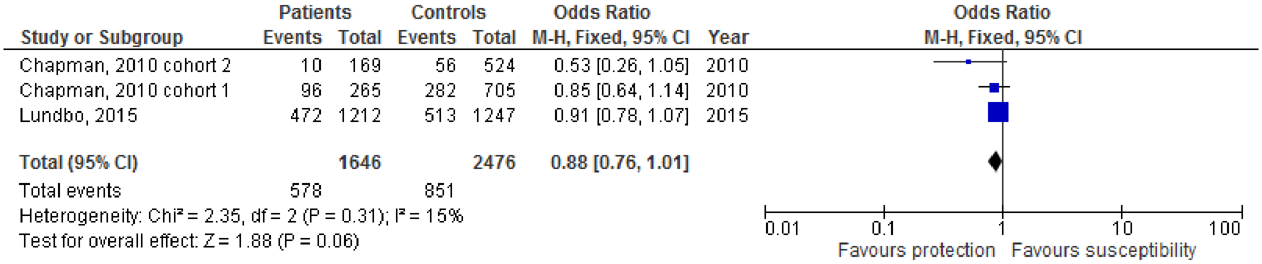


* HGVS representation: NC_000003.12(NFKBIZ):g.101850882A>C

Forest plot a.12 *PTPN22* – CT+TT genotypes of rs2476601*

| Study or Subgroup | Patients Events | Patients Total | Controls Events | Controls Total | Odds Ratio M-H, Random, 95% CI | Year |
|---|---|---|---|---|---|---|
| Chapman, 2006 | 66 | 286 | 131 | 803 | 1.54 [1.10, 2.15] | 2006 |
| Lundbo, 2015 | 207 | 1206 | 226 | 1247 | 0.94 [0.76, 1.15] | 2015 |
| **Total (95% CI)** | | **1492** | | **2050** | **1.18 [0.73, 1.92]** | |
| Total events | 273 | | 357 | | | |
| Heterogeneity: Tau² = 0.10; Chi² = 6.17, df = 1 (P = 0.01); I² = 84% | | | | | | |
| Test for overall effect: Z = 0.66 (P = 0.51) | | | | | | |

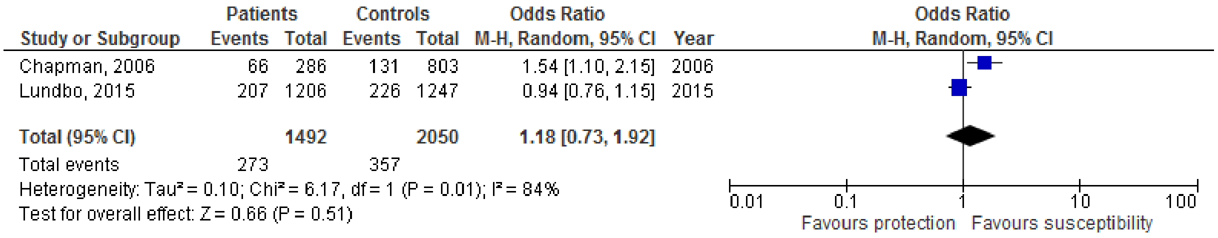


* HGVS representation: NM_015967.6(PTPN22):c.1858C>T (p.Arg620Trp)

Forest plot a.13 *TIRAP* - CT+TT genotypes (carriers variant allele) of rs8177374*

| Study or Subgroup | Patients Events | Patients Total | Controls Events | Controls Total | Odds Ratio M-H, Fixed, 95% CI | Year |
|---|---|---|---|---|---|---|
| Khor, 2007 cohort 1 | 45 | 191 | 214 | 751 | 0.77 [0.53, 1.12] | 2007 |
| Khor, 2007 cohort 2 | 11 | 36 | 115 | 361 | 0.94 [0.45, 1.98] | 2007 |
| Khor, 2007 cohort 3 | 3 | 164 | 25 | 423 | 0.30 [0.09, 1.00] | 2007 |
| Lundbo, 2015 | 318 | 1196 | 330 | 1242 | 1.00 [0.84, 1.20] | 2015 |
| Gowin, 2018 | 4 | 14 | 4 | 49 | 4.50 [0.96, 21.12] | 2018 |
| **Total (95% CI)** | | **1601** | | **2826** | **0.94 [0.80, 1.09]** | |
| Total events | 381 | | 688 | | | |
| Heterogeneity: Chi² = 8.97, df = 4 (P = 0.06); I² = 55% | | | | | | |
| Test for overall effect: Z = 0.82 (P = 0.41) | | | | | | |

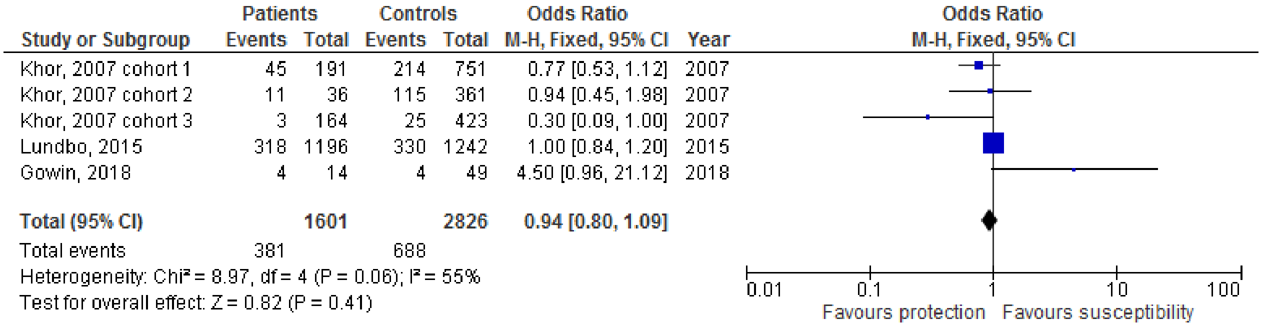


* HGVS representation: NM_001039661.1(TIRAP):c.539C>T (p.Ser180Leu)

Forest plot a.14 *TLR2* - GA+AA genotypes (RQ+QQ) of rs5743708*

| Study or Subgroup | Patients Events | Patients Total | Controls Events | Controls Total | Odds Ratio M-H, Random, 95% CI | Year |
|---|---|---|---|---|---|---|
| Moens, 2007 | 6 | 99 | 9 | 178 | 1.21 [0.42, 3.51] | 2007 |
| Yuan, 2008 | 3 | 85 | 27 | 409 | 0.52 [0.15, 1.75] | 2008 |
| van Well, 2013 | 9 | 82 | 100 | 1141 | 1.28 [0.62, 2.64] | 2013 |
| Tellería-Orriols, 2014 | 68 | 114 | 17 | 66 | 4.26 [2.19, 8.30] | 2014 |
| Gowin, 2017 | 2 | 14 | 3 | 49 | 2.56 [0.38, 17.06] | 2017 |
| **Total (95% CI)** | | **394** | | **1843** | **1.59 [0.74, 3.43]** | |
| Total events | 88 | | 156 | | | |
| Heterogeneity: Tau² = 0.48; Chi² = 11.94, df = 4 (P = 0.02); I² = 66% | | | | | | |
| Test for overall effect: Z = 1.18 (P = 0.24) | | | | | | |

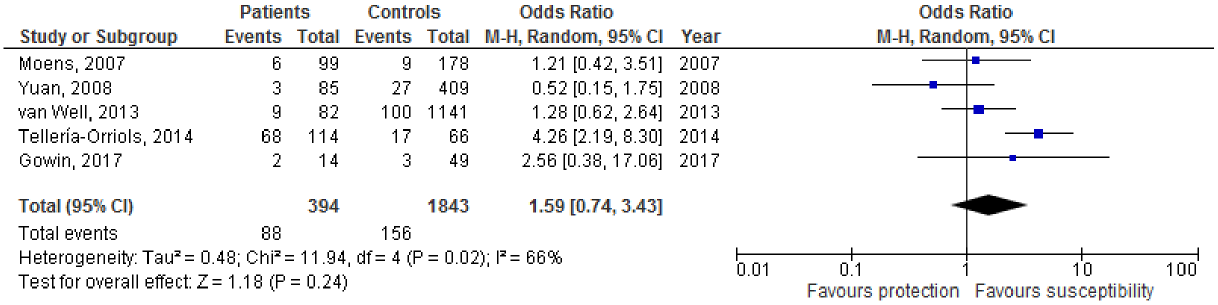


* HGVS representation: NM_003264.4(TLR2):c.2258G>A (p.Arg753Gln)

Forest plot a.15 *TLR4* - AG+GG genotypes (D299G+G299G) of rs4986790*

| Study or Subgroup | Patients Events | Patients Total | Controls Events | Controls Total | Odds Ratio M-H, Fixed, 95% CI | Year |
|---|---|---|---|---|---|---|
| Moens, 2007 | 15 | 99 | 17 | 178 | 1.69 [0.80, 3.55] | 2007 |
| Yuan, 2008 | 3 | 85 | 45 | 409 | 0.30 [0.09, 0.98] | 2008 |
| van Well, 2013 | 7 | 80 | 140 | 1141 | 0.69 [0.31, 1.52] | 2013 |
| Tellería-Orriols, 2014 | 20 | 114 | 6 | 66 | 2.13 [0.81, 5.60] | 2014 |
| Gowin, 2017 | 2 | 14 | 8 | 49 | 0.85 [0.16, 4.57] | 2017 |
| **Total (95% CI)** | | **392** | | **1843** | **0.96 [0.65, 1.42]** | |
| Total events | 47 | | 216 | | | |
| Heterogeneity: Chi² = 9.27, df = 4 (P = 0.05); I² = 57% | | | | | | |
| Test for overall effect: Z = 0.20 (P = 0.84) | | | | | | |

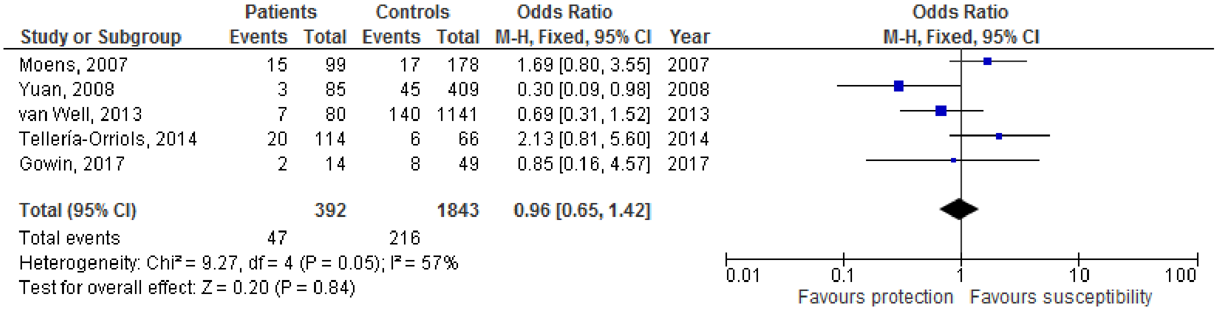


* HGVS representation: NM_138554.4(TLR4):c.896A>G (p.Asp299Gly)

Forest plot a.16 *TLR9* – CC genotype (wildtype) of rs352140*

| Study or Subgroup | Patients Events | Patients Total | Controls Events | Controls Total | Odds Ratio M-H, Fixed, 95% CI | Year |
|---|---|---|---|---|---|---|
| Sanders, 2011 | 17 | 80 | 66 | 392 | 1.33 [0.73, 2.42] | 2011 |
| Gowin, 2017 | 2 | 14 | 7 | 49 | 1.00 [0.18, 5.46] | 2017 |
| **Total (95% CI)** | | **94** | | **441** | **1.29 [0.73, 2.26]** | |
| Total events | 19 | | 73 | | | |
| Heterogeneity: Chi² = 0.10, df = 1 (P = 0.75); I² = 0% | | | | | | |
| Test for overall effect: Z = 0.88 (P = 0.38) | | | | | | |

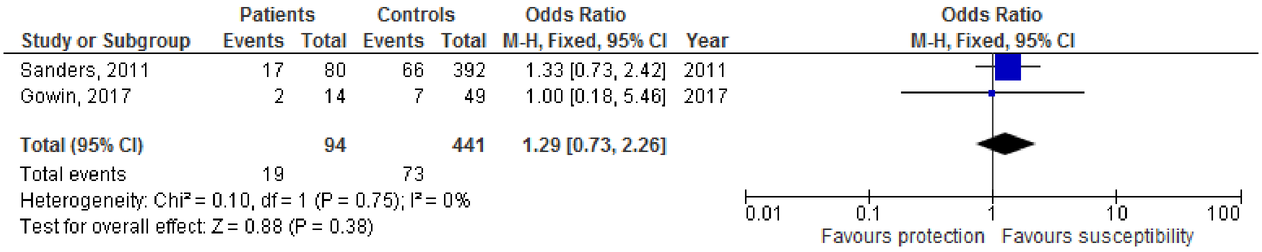


* HGVS representation: NC_000003.12(TLR9):g.52222681C>A

*† Events are amount of patients or controls with reference genotype (in plot title)*

### *b. Outcome studies †*

Forest plot b.1 on mortality and *MBL2* – variant alleles of rs1800450, rs1800451 or rs5030737 (O/O genotype)*

| Study or Subgroup | Deceased Events | Deceased Total | Survivors Events | Survivors Total | Odds Ratio M-H, Fixed, 95% CI | Year |
|---|---|---|---|---|---|---|
| Endeman, 2008 | 2 | 10 | 2 | 50 | 6.00 [0.74, 48.90] | 2008 |
| Brouwer, 2013 | 0 | 25 | 11 | 274 | 0.45 [0.03, 7.85] | 2013 |
| Mills, 2015 | 2 | 45 | 11 | 200 | 0.80 [0.17, 3.74] | 2015 |
| **Total (95% CI)** | | **80** | | **524** | **1.13 [0.40, 3.22]** | |
| Total events | 4 | | 24 | | | |
| Heterogeneity: Chi² = 3.03, df = 2 (P = 0.22); I² = 34% | | | | | | |
| Test for overall effect: Z = 0.23 (P = 0.82) | | | | | | |

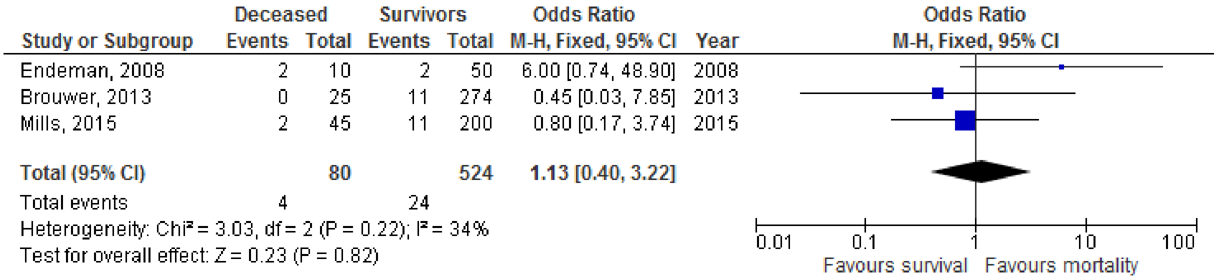


* HGVS representations: rs1800450; (NM_000242.2(MBL2):c.161G>A (p.Gly54Asp). rs1800451; NM_000242.2(MBL2):c.170G>A (p.Gly57Glu); rs5030737; NM_000242.2(MBL2):c.154C>T (p.Arg52Cys)

*† Events are amount of patients with reference genotype (in plot title)*
